# Boundedness of Complements for Log Calabi–Yau Threefolds

**DOI:** 10.1007/s42543-022-00057-x

**Published:** 2023-02-07

**Authors:** Guodu Chen, Jingjun Han, Qingyuan Xue

**Affiliations:** 1 Institute for Theoretical Sciences, Westlake University, Hangzhou, 310024 Zhejiang China; 2 Shanghai Center for Mathematical Sciences, Fudan University, Shanghai, 200438 China; 3 Department of Mathematics, The University of Utah, Salt Lake City, UT 84112 USA

**Keywords:** Complements, Log Calabi–Yau pairs, Fano varieties, 14E30, 14J45, 14J17

## Abstract

In this paper, we study the theory of complements, introduced by Shokurov, for Calabi–Yau type varieties with the coefficient set [0, 1]. We show that there exists a finite set of positive integers $\mathcal {N}$, such that if a threefold pair $(X/Z\ni z,B)$ has an $\mathbb {R}$-complement which is klt over a neighborhood of *z*, then it has an *n*-complement for some $n\in \mathcal {N}$. We also show the boundedness of complements for $\mathbb {R}$-complementary surface pairs.

## Introduction

We work over the field of complex numbers $\mathbb {C}$.

The theory of complements (for Fano varieties) was introduced by Shokurov when he proved the existence of flips for threefolds [45]. It originates from his earlier work on anti-canonical systems on Fano threefolds [44]. The boundedness of complements [4, 26, 47] played an important role in various contexts in the study of Fano varieties, including the solution of the Borisov–Alexeev–Borisov conjecture (boundedness of Fano varieties) [4, 5] and the Yau–Tian–Donaldson conjecture (the existence of Kähler–Einstein metrics on log Fano pairs) [8, 40, 49]. We refer the reader to [9–11, 13, 14, 16, 25, 26, 38] and references therein for more recent progress and applications.

According to the minimal model program, varieties of general type, Fano varieties and Calabi–Yau varieties form three fundamental classes in birational geometry and are building blocks of algebraic varieties. In this paper, we study the theory of complements for Calabi–Yau type varieties with the coefficient set [0, 1] in dimensions 2 and 3. Note that Calabi–Yau type varieties form a large class of varieties which includes both Fano varieties and Calabi–Yau varieties. For Calabi–Yau varieties, since the boundedness of complements implies the boundedness of the non-vanishing index of $K_X$, we expect that the theory of complements will play an important role in the study of Calabi–Yau varieties, including the boundedness of Calabi–Yau varieties. We also remark that replacing a coefficient set which satisfies the descending chain condition (DCC) with the set [0, 1] is considered as a very hard problem in the theory of complements.

Our first main result is the boundedness of complements for threefold pairs.

### Theorem 1.1

Let *l* be a positive integer. Then, there exists a finite set of positive integers $\mathcal {N}$ depending only on *l* satisfying the following.

Assume that $(X/Z\ni z,B)$ is a threefold pair which has an $\mathbb {R}$-complement that is klt over a neighborhood of *z*. Then, $(X/Z\ni z,B)$ has an *n*-complement for some $n\in \mathcal {N}$ such that $l\mid n$.

Theorem 1.1 fails if we remove the assumption “klt over a neighborhood of *z*”; see [47, Example 11]. However, if we require the coefficients of the boundaries to lie in a set $\Gamma \subseteq [0,1]$ such that $\Gamma \cap \mathbb {Q}$ is DCC, then we can remove the klt assumption.

### Theorem 1.2

Let *l* be a positive integer, and $\Gamma \subseteq [0,1]$ a set such that $\Gamma \cap \mathbb {Q}$ is DCC. Then, there exists a finite set of positive integers $\mathcal {N}$ depending only on *l* and $\Gamma$ satisfying the following.

Assume that $(X/Z\ni z,B)$ is an $\mathbb {R}$-complementary threefold pair such that *X* is of Calabi–Yau type over a neighborhood of *z* and $B\in \Gamma$. Then, $(X/Z\ni z,B)$ has an *n*-complement for some $n\in \mathcal {N}$ such that $l\mid n$.

Here, we say that *X* is *of Calabi–Yau type* over a neighborhood of *z*, if there exists a boundary *C* on *X* such that (*X*, *C*) is klt and $K_X+C\sim _{\mathbb {R},Z}0$ over a neighborhood of *z*; see Definition 7.1.

Our last main result is the boundedness of complements for surface pairs where we do not require the pair has a klt $\mathbb {R}$-complement nor ${\Gamma }\cap \mathbb {Q}$ is DCC. Theorem 1.3 completely answers a question of Shokurov [46, 1.3 Conjecture on complements] for surfaces.

### Theorem 1.3

Let *l* be a positive integer. Then, there exists a finite set of positive integers $\mathcal {N}$ depending only on *l* satisfying the following.

Assume that $(X/Z\ni z,B)$ is an $\mathbb {R}$-complementary surface pair. Then, $(X/Z\ni z,B)$ has an *n*-complement for some $n\in \mathcal {N}$ such that $l\mid n$.

**Sketch of proofs.** We now sketch the proofs of Theorems 1.1 and 1.3. For convenience, in what follows, we will assume that $l=1$ and (*X*, *B*) is a $\mathbb {Q}$-factorial klt log Calabi–Yau pair, that is, $Z=z=\{pt\}$, (*X*, *B*) is $\mathbb {Q}$-factorial klt and $K_X+B\sim _\mathbb {R}0$.

We first sketch the proof of Theorem 1.3. If *X* is of Fano type, then (*X*, *B*) is $\mathcal {N}_1$-complementary for some finite set of positive integers $\mathcal {N}_1$ by Theorem 2.19; here, (*X*, *B*) being $\mathcal {N}_1$-complementary means that (*X*, *B*) is *n*-complementary for some $n\in \mathcal {N}_1$ (see Definition 2.12). Thus, we may assume that *X* is not of Fano type and $\kappa (X,B-B_{\Phi _1})\le 1$, where $\Phi _1:=\Gamma (\mathcal {N}_1,\{0,1\})$ is a hyperstandard set and $B_{\Phi _1}$ is a $\mathbb {Q}$-divisor with coefficients in $\Phi _1$ such that $0 \le B_{\Phi _1} \le B$ (see Definition 2.1). Suppose that $\kappa (X,B-B_{\Phi _1})=\kappa (X,B-B_{\Phi _2})=1$, where $\mathcal {N}_2$ is a finite set of positive integers given by Theorem 2.20 and $\Phi _2:=\Gamma (\mathcal {N}_1\cup \mathcal {N}_2,\{0,1\})$. In this case, we claim that (*X*, *B*) is $\mathcal {N}_2$-complementary. Indeed, although *X* is not of Fano type, by Lemma 2.15 we can still run an MMP on $-(K_X+B_{\Phi _2})$ and get a good minimal model $X'$ such that $-(K_{X'}+B_{\Phi _2}')$ is semi-ample and hence defines a contraction $\pi ':X'\rightarrow Z'$, where $D'$ denotes the strict transform of *D* on $X'$ for any $\mathbb {R}$-divisor *D* on *X*. Then, we run an MMP on $-(K_{X'}+B_{\Phi _1}')$ over $Z'$ and reach a model $X''$ on which $-(K_{X''}+B_{\Phi _1}'')$ is semi-ample over $Z'$, where $D''$ denotes the strict transform of *D* on $X''$ for any $\mathbb {R}$-divisor *D* on *X*. As $\kappa (X,B-B_{\Phi _1})=\kappa (X,B-B_{\Phi _2})=1$, the natural morphism $\pi '':X''\rightarrow Z'$ is the contraction defined by $-(K_{X''}+B_{\Phi _1}'')$ over $Z'$. By the similar arguments as in [4, Proposition 6.3] and using Effective Adjunction [43, Conjecture 7.13.3 and Theorem 8.1], there exists a positive integer *p* which only depends on $\Phi _1$ such that $p(K_{X''}+B_{\Phi _1}'')\sim p(\pi '')^*(K_{Z'}+B_{Z'}^{(1)}+\textbf{M}_{\pi '',Z'})$ and $p\textbf{M}_{\pi ''}$ is base point free, where $B_{Z'}^{(1)}$ and $\textbf{M}_{\pi ''}$ are given by the canonical bundle formula for $(X'',B_{\Phi _1}'')$ over $Z'$ in Proposition 3.3. It follows that $p(K_{X'}+B_{\Phi _2}')\sim p(\pi ')^*(K_{Z'}+B_{Z'}^{(2)}+\textbf{M}_{\pi ',Z'})$ and $p\textbf{M}_{\pi '}$ is base point free, where $B_{Z'}^{(2)}$ and $\textbf{M}_{\pi '}$ are given by the canonical bundle formula for $(X',B_{\Phi _2}')$ over $Z'$. We may assume that $p\mid n$ for any $n \in \mathcal {N}_2$. As $p\textbf{M}_{\pi '}$ is base point free, one can find an effective $\mathbb {Q}$-divisor $M_{Z'}$ such that $pM_{Z'}\sim p\textbf{M}_{\pi ',Z'}$, $M_{Z'}$ has no common components with $B_{Z'}^{(2)}$, and $(Z',B_{Z'}^{(2)}+M_{Z'})$ has an *n*-complement for some $n\in \mathcal {N}_2$. Then, we can lift this complement to *X* and get an *n*-complement of (*X*, *B*); see Proposition 3.5. If $\kappa (X,B-B_{\Phi _2})=0$, then we can easily show that $n_0(K_X+B)\sim 0$ for some positive integer $n_0$ which only depends on $\Phi _2$; see Lemma 2.18. Hence, $\mathcal {N}_1\cup \mathcal {N}_2\cup \{n_0\}$ has the required property.

Now, we sketch the proof of Theorem 1.1. The main strategy is similar. One of the key steps is to construct a positive integer $n_0$ and finite sets of positive integers $\mathcal {N}_{i}$ ($i=1,2,3$) such that

1. if $\kappa (X,B-B_{\Phi _1})=3$, then (*X*, *B*) is $\mathcal {N}_1$-complementary,
2. if $\kappa (X,B-B_{\Phi _1})=\kappa (X,B-B_{\Phi _2})=2$, then (*X*, *B*) is $\mathcal {N}_2$-complementary,
3. if $\kappa (X,B-B_{\Phi _2})=\kappa (X,B-B_{\Phi _3})=1$, then (*X*, *B*) is $\mathcal {N}_3$-complementary, and
4. if $\kappa (X,B-B_{\Phi _3})=0$, then (*X*, *B*) is $n_0$-complementary,

where $\Phi _i:=\Gamma (\bigcup _{j=1}^i\mathcal {N}_j,\{0,1\})$ for any $1\le i\le 3$; see Sect. 6 for the details. However, there are some issues when we construct these finite sets. One issue is that when we apply the canonical bundle formula, Effective Adjunction is still open when the relative dimension is $\ge 2$. But in our setting we can give a positive answer to Effective Adjunction; see Proposition 3.4 for the details. On the other hand, there is also an issue when we try to lift complements from lower dimensional varieties. More precisely, it may happen that some components of ${\text {Supp}}B$ have images of codimension $\ge 2$ in *Z*. Therefore, we must lift complements more carefully; see Proposition 3.5 and Sect. 6 for the details.

*Structure of the paper.* We outline the organization of the paper. In Sect. 2, we introduce some notation and tools which will be used in this paper, and prove certain basic results. In Sect. 3, we recall the canonical bundle formula, some well-known results, as well as some new results. In Sect. 4, we prove the boundedness of complements for sdlt curves. In Sect. 5, we prove Theorem 1.3. In Sect. 6, we prove Theorem 1.1. In Sect. 7, we prove Theorem 1.2.

## Preliminaries

### Arithmetic of Sets

#### Definition 2.1

(1) We say that a set $\Gamma \subseteq [0,1]$ satisfies the *descending chain condition* (DCC) if any decreasing sequence $a_1\ge a_2\ge \cdots$ in $\Gamma$ stabilizes. We say that $\Gamma$ satisfies the *ascending chain condition* (ACC) if any increasing sequence $a_1\le a_2\le \cdots$ in $\Gamma$ stabilizes.

(2) Suppose that $\mathfrak {R}\subseteq [0,1]\cap \mathbb {Q}$ is a finite set. We define

$$\begin{aligned} \Phi (\mathfrak {R}):=\left\{ 1-\frac{r}{l}\,\bigg | \,r\in \mathfrak {R},l\in \mathbb {Z}_{>0}\right\} \cap [0,1] \end{aligned}$$

to be the set of *hyperstandard multiplicities* associated to $\mathfrak {R}$ (cf. [4, 2.2]). We may say that $\Phi (\mathfrak {R})$ is the *hyperstandard set* associated to $\mathfrak {R}$. When we say $\Phi \subseteq [0,1]\cap \mathbb {Q}$ is a hyperstandard set, we mean that $\Phi =\Phi (\mathfrak {R})$ for some finite set $\mathfrak {R}\subseteq [0,1]\cap \mathbb {Q}$. We usually assume $0,1\in \mathfrak {R}$ without mentioning, so $\Phi (\{0,1\})\subseteq \Phi (\mathfrak {R})$.

(3) (cf. [47, Page 30]) Let $\mathcal {N}\subseteq \mathbb {Z}_{>0}$, $\mathfrak {R}\subseteq [0,1]\cap \mathbb {Q}$ be two finite sets, and $\Phi :=\Phi (\mathfrak {R})$. We define

$$\begin{aligned} \Gamma (\mathcal {N},\Phi (\mathfrak {R})):=\bigg \{1-\frac{r}{l}+\frac{1}{l}\sum _{n\in \mathcal {N}}\frac{m_n}{n+1} \, \bigg | \, r\in \mathfrak {R},l\in \mathbb {Z}_{>0},m_n\in \mathbb {Z}_{\ge 0}\bigg \}\cap [0,1]. \end{aligned}$$

By Remark 2.2 (1), $\Gamma (\mathcal {N},\Phi (\mathfrak {R}))$ is independent of the choice of $\mathfrak {R}$. Hence, we may write $\Gamma (\mathcal {N},\Phi )$ instead of $\Gamma (\mathcal {N},\Phi (\mathfrak {R}))$ for convenience. By Remark 2.2 (2), $\Gamma (\mathcal {N},\Phi )$ is a hyperstandard set. In particular, it is a DCC set whose only accumulation point is 1. Then, for any $b\in [0,1]$, we define

$$\begin{aligned} b_{\mathcal {N}\_\Phi }:=\max \left\{ b'| b'\le b,\, b'\in {\Gamma }(\mathcal {N},\Phi )\right\} . \end{aligned}$$

If $\mathcal {N}=\{n\}$ (respectively, $\mathcal {N}=\emptyset$), we may write $b_{n\_\Phi }$ (respectively, $b_{\Phi }$) rather than $b_{\mathcal {N}\_\Phi }$.

#### Remark 2.2

(1) If $\mathfrak {R}'\subseteq [0,1]\cap \mathbb {Q}$ is a finite set such that $\Phi (\mathfrak {R})=\Phi (\mathfrak {R}')$, then $\Gamma (\mathcal {N},\Phi (\mathfrak {R}))=\Gamma (\mathcal {N},\Phi (\mathfrak {R}')).$ Indeed, for any $r'\in \mathfrak {R}'$, there exist $r\in \mathfrak {R}$ and $l\in \mathbb {Z}_{>0}$ such that $r'=r/l$. Thus, $\Gamma (\mathcal {N},\Phi (\mathfrak {R}))\supseteq \Gamma (\mathcal {N},\Phi (\mathfrak {R}'))$, and the converse inclusion follows similarly.

(2) ${\Gamma }(\mathcal {N},\Phi )$ is the hyperstandard set associated to the following finite set:

$$\begin{aligned} \mathfrak {R}'' := \bigg \{r-\sum _{n\in \mathcal {N}}\frac{m_n}{n+1} \, \bigg | \, r\in \mathfrak {R},m_n\in \mathbb {Z}_{\ge 0}\bigg \}\cap [0,1]. \end{aligned}$$

Indeed,

$$\begin{aligned} \Phi (\mathfrak {R}'')&=\bigg \{1-\frac{r''}{l}\, \bigg | \, r''\in \mathfrak {R}'',l\in \mathbb {Z}_{>0}\bigg \}\cap [0,1]\\&= \bigg \{1-\frac{r}{l}+\frac{1}{l}\sum _{n\in \mathcal {N}}\frac{m_n}{n+1}\, \bigg | \, r\in \mathfrak {R},l\in \mathbb {Z}_{>0},m_n\in \mathbb {Z}_{\ge 0}\bigg \}\cap [0,1] \\ {}&= {\Gamma }(\mathcal {N},\Phi ). \end{aligned}$$

(3) If $\mathcal {N}_1$ and $\mathcal {N}_2$ are two finite sets of positive integers, then

$$\begin{aligned} \Gamma (\mathcal {N}_{1}\cup \mathcal {N}_2,\Phi )=\Gamma (\mathcal {N}_2,\Gamma (\mathcal {N}_1,\Phi )). \end{aligned}$$

Indeed, let

$$\begin{aligned} \mathfrak {R}_1 := \bigg \{r-\sum _{n\in \mathcal {N}_1}\frac{m_n}{n+1} \, \bigg | \, r\in \mathfrak {R},m_n\in \mathbb {Z}_{\ge 0}\bigg \}\cap [0,1]. \end{aligned}$$

Then, $\Gamma (\mathcal {N}_1,\Phi )=\Phi (\mathfrak {R}_1)$ by (2). Therefore,

$$\begin{aligned}&\Gamma (\mathcal {N}_2,\Gamma (\mathcal {N}_1,\Phi ))=\Gamma (\mathcal {N}_2,\Phi (\mathfrak {R}_1))\\&\quad =\bigg \{1-\frac{r_1}{l}+\frac{1}{l}\sum _{n\in \mathcal {N}_2}\frac{m_n}{n+1}\,\bigg | \,r_1\in \mathfrak {R}_1,l\in \mathbb {Z}_{>0},m_n\in \mathbb {Z}_{\ge 0}\bigg \}\cap [0,1]\\&\quad =\bigg \{1-\frac{1}{l}\bigg (r-\sum _{n'\in \mathcal {N}_1}\frac{m'_{n'}}{n'+1}\bigg )+\frac{1}{l}\sum _{n\in \mathcal {N}_2}\frac{m_n}{n+1}\, \bigg | \, r\in \mathfrak {R},l\in \mathbb {Z}_{>0},m'_{n'},m_n\in \mathbb {Z}_{\ge 0}\bigg \}\cap [0,1]\\&\quad =\bigg \{1-\frac{r}{l}+\frac{1}{l}\bigg (\sum _{n'\in \mathcal {N}_1}\frac{m'_{n'}}{n'+1}+\sum _{n\in \mathcal {N}_2}\frac{m_n}{n+1}\bigg )\, \bigg | \, r\in \mathfrak {R},l\in \mathbb {Z}_{>0},m_n,m'_{n'}\in \mathbb {Z}_{\ge 0}\bigg \}\cap [0,1]\\&\quad =\Gamma (\mathcal {N}_{1}\cup \mathcal {N}_2,\Phi ). \end{aligned}$$

The following lemma was observed by the second named author. It will play an important role in the proof of the main theorems.

#### Lemma 2.3

Assume that $\mathfrak {R}\subseteq [0,1]\cap \mathbb {Q}$ is a finite set, $\Phi :=\Phi (\mathfrak {R})$, and *n* is a positive integer such that $n\mathfrak {R}\subseteq \mathbb {Z}$. Then, for any $b\in [0,1]$, we have

$$\begin{aligned} \frac{\lfloor (n+1)\{b\}\rfloor }{n}+\lfloor b\rfloor \ge b_{n{\_}\Phi }. \end{aligned}$$

#### Proof

Without loss of generality, we may assume that $1>b=b_{n{\_}\Phi }=1-\frac{r}{l}+\frac{m}{l(n+1)}\in {\Gamma }(\{n\},\Phi )$ for some $r\in \mathfrak {R},l\in \mathbb {Z}_{>0}$ and $m\in \mathbb {Z}_{\ge 0}$. It suffices to show that

$$\begin{aligned} \bigg \lfloor 1-\frac{(n+1)r}{l}+\frac{m}{l}\bigg \rfloor \ge -\frac{nr}{l}+\frac{mn}{l(n+1)}. \end{aligned}$$

Suppose on the contrary that there exists an integer *k* such that

$$\begin{aligned} 1-\frac{(n+1)r}{l}+\frac{m}{l}<k \quad \text {and}\quad -\frac{nr}{l}+\frac{mn}{l(n+1)}>k-1. \end{aligned}$$

The first inequality above gives us that $l-lk+m<(n+1)r$, and thus $l-lk+m\le nr$ as $nr\in \mathbb {Z}$. Therefore, we have

$$\begin{aligned} \frac{mn}{n+1}> (k-1)l+nr\ge (k-1)l+l-lk+m=m, \end{aligned}$$

a contradiction. $$\square $$

#### Lemma 2.4

Let $\mathcal {N}$ be a finite set of positive integers, $\Phi$ a hyperstandard set, and $n\in \mathcal {N}$. Suppose that $b,b^+\in [0,1]$ such that $nb^+\in \mathbb {Z}$ and

$$\begin{aligned} nb^+\ge \lfloor (n+1)\{b_{\mathcal {N}\_\Phi }\}\rfloor +n\lfloor b_{\mathcal {N}\_\Phi }\rfloor . \end{aligned}$$

Then, $$nb^+\ge \lfloor (n+1)\{b\}\rfloor +n\lfloor b\rfloor $$.

#### Proof

If $b=1$, then $b^+=b_{\mathcal {N}\_\Phi }=1$ and there is nothing to prove.

If $b<1$, then $b_{\mathcal {N}\_\Phi }\le b<1$. It suffices to show that $\lfloor {(n+1)b}\rfloor = \lfloor {(n+1)b_{\mathcal {N}\_\Phi }}\rfloor$. Let

$$\begin{aligned} b':=\max \bigg \{\frac{l}{n+1}\, \bigg | \, \frac{l}{n+1}\le b, l\in \mathbb {Z}_{\ge 0}\bigg \}\in \Gamma (\mathcal {N},\Phi ). \end{aligned}$$

By the construction, $\lfloor {(n+1)b'}\rfloor =\lfloor {(n+1)b}\rfloor$, which implies that $\lfloor {(n+1)b}\rfloor =\lfloor {(n+1)b_{\mathcal {N}\_\Phi }}\rfloor$ as $b'\le b_{\mathcal {N}\_\Phi }\le b$. $\square$

### Divisors

Let $\mathbb {F}$ be either the rational number field $\mathbb {Q}$ or the real number field $\mathbb {R}$. Let *X* be a normal variety and ${\text {WDiv}}(X)$ the free abelian group of Weil divisors on *X*. Then, an $\mathbb {F}$-*divisor* is defined to be an element of ${\text {WDiv}}(X)_\mathbb {F}:={\text {WDiv}}(X)\otimes \mathbb {F}$.

A *b-divisor* on *X* is an element of the projective limit

$$\begin{aligned} {\textbf {WDiv}}(X)=\lim _{Y\rightarrow X}{\text {WDiv}}(Y), \end{aligned}$$

where the limit is taken over all the pushforward homomorphisms $f_*:{\text {WDiv}}(Y)\rightarrow {\text {WDiv}}(X)$ induced by proper birational morphisms $f:Y\rightarrow X$. In other words, a b-divisor *D* on *X* is a collection of Weil divisors ${\textbf {D}}_Y$ on higher models *Y* of *X* that are compatible with respect to pushforward. The divisor ${\textbf {D}}_Y$ is called the *trace* of ${\textbf {D}}$ on the birational model *Y*. A *b*-$\mathbb {F}$-*divisor* is defined to be an element of ${\textbf {WDiv}}(X)\otimes \mathbb {F}$, and the trace of a b-$\mathbb {F}$-divisor is defined similarly.

The *Cartier closure* of an $\mathbb {F}$-Cartier $\mathbb {F}$-divisor *D* on *X* is the b-$\mathbb {F}$-divisor $\overline{D}$ with trace $\overline{D}_Y=f^*D$ for any proper birational morphism $f:Y\rightarrow X$. A b-$\mathbb {F}$-divisor ${\textbf {D}}$ on *X* is *b*-$\mathbb {F}$-*Cartier* if ${\textbf {D}}=\overline{D_{Y}}$ where $\overline{D_Y}$ is an $\mathbb {F}$-Cartier $\mathbb {F}$-divisor on a birational model *Y* of *X*; in this situation, we say ${\textbf {D}}$ *descends to* *Y*. Moreover, if $D_Y$ is a Cartier divisor, then we say $\textbf{D}$ is *b-Cartier*. Let $X\rightarrow Z$ be a projective morphism. Then, a b-$\mathbb {F}$-divisor is *nef* (respectively, *base point free*) *over* *Z* if it descends to a nef (respectively, base point free) over *Z* $\mathbb {F}$-divisor on some birational model of *X*.

Assume that $\Gamma \subseteq [0,1]$ is a set, and $B:=\sum b_iB_i$, $B':=\sum b_i'B_i$ are two $\mathbb {R}$-divisors on *X*, where $B_i$ are prime divisors. By $B\in \Gamma$ we mean $b_i\in \Gamma$ for any *i*. We define $\lfloor B\rfloor :=\sum \lfloor b_i\rfloor B_i,\, \{B\}:=\sum \{b_i\}B_i,\, \Vert B\Vert :=\max \{|b_i|\}$, and $B\wedge B':=\sum \min \{b_i,b_i'\}B_i$. Assume that $\mathcal {N}$ is a finite set of positive integers and $\Phi$ is a hyperstandard set. We define

$$\begin{aligned} B_{\mathcal {N}\_\Phi }:=\sum (b_i)_{\mathcal {N}\_\Phi }B_i. \end{aligned}$$

If $\mathcal {N}=\{n\}$ (respectively, $\mathcal {N}=\emptyset$), we may write $B_{n\_\Phi }$ (respectively, $B_{\Phi }$) instead of $B_{\mathcal {N}\_\Phi }$.

#### Definition 2.5

(1) We say $\pi : X \rightarrow Z$ is a *contraction* if *X* and *Z* are normal quasi-projective varieties, $\pi$ is a projective morphism, and $\pi _*\mathcal {O}_X = \mathcal {O}_Z$.

(2) We say that a birational map $\phi : X \dashrightarrow Y$ is a *birational contraction* if $\phi$ is projective and $\phi ^{-1}$ does not contract any divisors.

#### Lemma 2.6

Suppose that $\tau :Z''\rightarrow Z'$ and $Z'\rightarrow Z$ are contractions. Suppose that $H''$ (respectively, $H'$) is an $\mathbb {R}$-Cartier $\mathbb {R}$-divisor on $Z''$ (respectively, $Z'$) which is ample over $Z'$ (respectively, *Z*). Then, $\epsilon H''+\tau ^*H'$ is ample over *Z* for any $0<\epsilon \ll 1$.

#### Proof

Pick any closed point $z\in Z$. Let $Z'_z:=Z'\times _Z\{z\}$, $Z''_z:=Z''\times _Z\{z\}$, $H'_z:=H'|_{Z'_z}$, and $H''_z:=H''|_{Z''_z}$. By assumption $H''_z$ is ample over $Z'_z$ and $H'_z$ is ample. According to [36, Proposition 1.45], $\epsilon _zH''_z+(\tau |_{Z''_z})^*H'_z$ is ample for any $0<\epsilon _z\ll 1$. In particular, $\epsilon _z H''+\tau ^*H'$ is ample over *z*. It follows that $\epsilon _z H''+\tau ^*H'$ is ample over some neighborhood of *z* by [37, Theorem 1.2.17]. Since *Z* is quasi-compact, the lemma follows. $\square$

### Generalized Pairs and Singularities

In this paper, we usually discuss the (sub-)pair in the relative setting $(X/Z\ni z,B)$; we refer the reader to [11, §2] (cf. [6, 36]). Moreover, if the (sub-)pair $(X/Z\ni z,B)$ is (sub-)lc over *z* for any $z\in Z$, then we say (*X*/*Z*, *B*) is (sub-)lc.

Here, we briefly discuss the analogous concepts for generalized pairs, and refer the reader to [7, 22, 24] for further details.

#### Definition 2.7

A *generalized pair* (*g-pair* for short) $(X/Z,B+\textbf{M})$ consists of a contraction $X\rightarrow Z$, an effective $\mathbb {R}$-divisor *B* on *X*, and a nef/*Z* b-$\mathbb {R}$-divisor $\textbf{M}$ on *X*, such that $K_X+B+\textbf{M}_X$ is $\mathbb {R}$-Cartier.

Let $(X/Z,B+\textbf{M})$ be a g-pair and $f:W \rightarrow X$ a log resolution of $(X,{\text {Supp}}B)$ to which $\textbf{M}$ descends. We may write

$$\begin{aligned} K_{W}+B_W+\textbf{M}_W=f^*(K_X+B+\textbf{M}_X) \end{aligned}$$

for some $$\mathbb {R}$$-divisor $$ B_W$$ on *W*. Let *E* be a prime divisor on *W*. The *log discrepancy* of *E* with respect to $$(X,B+\textbf{M})$$ is defined as

$$\begin{aligned} a(E,X,B+\textbf{M}):=1-{\text {mult}}_EB_W. \end{aligned}$$

We say $(X/Z,B+\textbf{M})$ is *generalized lc* or *glc* (respectively, *generalized klt* or *gklt*) if $a(E,X,B+\textbf{M})\ge 0$ (respectively, $>0$) for any prime divisor *E* over *X*.

We say that two g-pairs $(X/Z,B+\textbf{M})$ and $(X'/Z,B'+\textbf{M}')$ are *crepant* if *X* is birational to $X'$, $\textbf{M}=\textbf{M}'$, and $f^*(K_X+B+\textbf{M}_X)=(f')^*(K_{X'}+B'+\textbf{M}'_{X'})$ for some common resolution $f:W\rightarrow X$ and $f':W\rightarrow X'$. We also call $(X'/Z,B'+\textbf{M})$ a *crepant model* of $(X/Z,B+\textbf{M})$.

#### Lemma 2.8

Let *d* be a positive integer and $\Gamma \subseteq [0,1]$ a DCC set. Then, there is a positive real number $\epsilon$ depending only on *d* and $\Gamma$ satisfying the following. Assume that (*X*, *B*) is a projective klt pair of dimension *d* such that $K_X+B\sim _{\mathbb {R}}0$ and $B\in \Gamma$. Then, (*X*, *B*) is $\epsilon$-lc.

#### Proof

The lemma follows from [4, Lemma 2.48]. $\square$

#### Definition 2.9

Let $X\rightarrow Z$ be a contraction and *D* an $\mathbb {R}$-Cartier $\mathbb {R}$-divisor on *X*. We denote by $\kappa (X,D)$ and $\kappa (X/Z,D)$ the *Iitaka dimension* and *relative Iitaka dimension* of *D* respectively; see [41, II, §3.b and §3.c].

#### Definition 2.10

Let $X\rightarrow Z$ be a contraction, *D* an $\mathbb {R}$-Cartier $\mathbb {R}$-divisor on *X*, and $\phi :X\dashrightarrow Y$ a birational contraction of normal quasi-projective varieties over *Z*. We say that *Y* is a *good minimal model* of *D* over *Z*, if $\phi$ is *D*-negative, $D_Y$ is semi-ample over *Z*, and *Y* is $\mathbb {Q}$-factorial, where $D_Y$ is the strict transform of *D* on *Y*.

#### Lemma 2.11

Let $X\rightarrow Z$ be a contraction, and $D_2\ge D_1$ two effective $\mathbb {R}$-Cartier $\mathbb {R}$-divisors on *X*. Suppose that $X\dashrightarrow X'$ is a sequence of steps of the $D_1$-MMP over *Z*. Let $D_2'$ be the strict transform of $D_2$ on $X'$. Then, $\kappa (X/Z,D_2)=\kappa (X'/Z,D_2')$.

#### Proof

Pick a positive real number $\epsilon$ such that $X\dashrightarrow X'$ is also a sequence of steps of the $(D_1+\epsilon D_2)$-MMP over *Z*. As ${\text {Supp}}(D_1+\epsilon D_2)={\text {Supp}}D_2$, one can see that

$$\begin{aligned} \kappa (X'/Z,D_2')=\kappa (X'/Z,D_1'+\epsilon D_2')=\kappa (X/Z,D_1+\epsilon D_2)=\kappa (X/Z,D_2), \end{aligned}$$

where $D_1'$ is the strict transform of $D_1$ on $X'$. $\square$

### Complements

#### Definition 2.12

We say that a pair $(X/Z\ni z,B^+)$ is an $\mathbb {R}$-*complement* of $(X/Z\ni z,B)$ if $(X,B^+)$ is lc, $B^+\ge B$, and $K_X+B^+\sim _\mathbb {R}0$ over a neighborhood of *z*. In addition if $(X,B^+)$ is klt over a neighborhood of *z*, then we call $(X/Z\ni z,B^+)$ a *klt* $\mathbb {R}$-*complement* of $(X/Z\ni z,B)$.

Let *n* be a positive integer. An *n*-*complement* of $(X/Z\ni z,B)$ is a pair $(X/Z\ni z,B^+),$ such that over some neighborhood of *z*,  we have

1. $$(X,B^+)$$ is lc,
2. $$n(K_X+B^+)\sim 0,$$ and
3. $$nB^+\ge n\lfloor B\rfloor +\lfloor (n+1)\{B\}\rfloor .$$

We say that $(X/Z\ni z, B^{+})$ is a *monotonic* *n*-*complement* of $(X/Z\ni z, B)$ if we additionally have $B^{+}\ge B$.

Let $\mathcal {N}$ be a non-empty set of positive integers. We say that $(X/Z\ni z,B)$ is $\mathcal {N}$-*complementary* (respectively, *n*-*complementary*, $\mathbb {R}$-*complementary*) if $(X/Z\ni z,B)$ has an *m*-complement (respectively, *n*-complement, $\mathbb {R}$-complement) for some $m\in \mathcal {N}$. If $(X/Z\ni z,B)$ has an $\mathbb {R}$-complement (respectively, *n*-complement) for any $z\in Z$, then we say that (*X*/*Z*, *B*) is $\mathbb {R}$-*complementary* (respectively, *n*-*complementary*).

Note that if $z' \in \bar{z}$ is a closed point and $(X/Z\ni z',B^+)$ is an $\mathbb {R}$-complement (respectively, an *n*-complement) of $(X/Z\ni z',B)$, then $(X/Z\ni z,B^+)$ is an $\mathbb {R}$-complement (respectively, an *n*-complement) of $(X/Z\ni z,B)$. Hence, when proving the existence of complements we may assume that $z \in Z$ is a closed point.

The following lemma is well-known to experts. We will use the lemma frequently without citing it in this paper.

#### Lemma 2.13

(cf. [4, 6.1)], [11, Lemma 2.11]) Let *n* be a positive integer. Assume that $(X/Z\ni z,B)$ is a pair, $\psi :X\dashrightarrow X'$ is a birational contraction over *Z*, and $B'$ is the strict transform of *B* on $X'$.

1. If $(X/Z\ni z,B)$ is $\mathbb {R}$-complementary (respectively, *n*-complementary), then so is $(X'/Z\ni z,B')$.
2. Suppose $\psi$ is $-(K_X+B)$-non-positive. If $(X'/Z\ni z,B')$ has an $\mathbb {R}$-complement (respectively, a monotonic *n*-complement), then so does $(X/Z\ni z,B)$.

The following lemma is an easy consequence of Lemmas 2.3 and 2.4.

#### Lemma 2.14

Let $\mathcal {N}\subseteq \mathbb {Z}_{>0},$ $\mathfrak {R}\subseteq [0,1]\cap \mathbb {Q}$ be two finite sets, $\Phi :=\Phi (\mathfrak {R})$, and *n* a positive integer such that $n\mathfrak {R}\subseteq \mathbb {Z}_{\ge 0}$. Assume that $(X/Z\ni z,B)$ is a pair.

1. If $(X/Z\ni z,B_{\mathcal {N}\_\Phi })$ is $\mathcal {N}$-complementary, then so is $(X/Z\ni z,B)$.
2. Any *n*-complement of $(X/Z\ni z,B)$ is a monotonic *n*-complement of $(X/Z\ni z,B_{n\_\Phi })$.

We will use the following lemma frequently in this paper.

#### Lemma 2.15

Let $\Phi \subseteq [0,1]\cap \mathbb {Q}$ be a hyperstandard set. Assume that $(X/Z\ni z,B)$ is a pair which is an $\mathbb {R}$-complement of itself. If either

- $\dim X=2$ and *X* is $\mathbb {Q}$-factorial, or
- $\dim X=3$ and (*X*, *B*) is dlt over a neighborhood of *z*,

then $-(K_X+B_\Phi )$ has a good minimal model over a neighborhood of *z*.

#### Proof

According to [11, Lemma 4.2], possibly shrinking *Z* near *z*, there exist a positive real number *u* and a surface (respectively, threefold) pair $(X,\Delta )$, such that the coefficients of $\Delta$ are at most 1 (respectively, $(X,\Delta )$ is dlt) and $-u(K_X+B_\Phi )\sim _{\mathbb {R},Z} K_X+\Delta$. In both cases, we can run an MMP on $K_X+\Delta$ over *Z* and reach a good minimal model $X'$ over *Z* by [18, 32, 48]. It is clear that $X'$ is a good minimal model of $-(K_X+B_\Phi )$ as $-u(K_X+B_\Phi )\sim _{\mathbb {R},Z} K_X+\Delta$. This finishes the proof. $\square$

### Boundedness of Complements

We propose a conjecture on the boundedness of complements and collect some useful results.

For a positive integer *l* and a non-empty set $\mathcal {N}\subseteq \mathbb {Z}_{>0}$, we say $\mathcal {N}$ is divisible by *l*, denoted by $l\mid \mathcal {N}$, if $l\mid n$ for any $n\in \mathcal {N}$.

#### Conjecture 2.16

Let *d*, *l* be two positive integers and $\Phi \subseteq [0,1]\cap \mathbb {Q}$ a hyperstandard set. Then, there exists a finite set of positive integers $\mathcal {N}$ divisible by *l* depending only on *d*, *l* and $\Phi$ satisfying the following.

Assume that $(X/Z\ni z,B)$ is a pair of dimension *d* such that $(X/Z\ni z,B_{\mathcal {N}\_\Phi })$ has an $\mathbb {R}$-complement which is klt over a neighborhood of *z*. Then, $(X/Z\ni z,B)$ is $\mathcal {N}$-complementary.

#### Remark 2.17

1. In Conjecture 2.16, we do not assume $(X/Z\ni z,B)$ is lc.
2. One can not remove the klt assumption in Conjecture 2.16 when $d\ge 3$; see [47, Example 11]. However, we will show Conjecture 2.16 for $\mathbb {R}$-complementary surface pairs without the klt assumption; see Theorem 1.3.

#### Lemma 2.18

Let $\Phi \subseteq [0,1]\cap \mathbb {Q}$ be a hyperstandard set. Then, there exists a positive integer *n* depending only on $\Phi$ satisfying the following.

Assume that (*X*, *B*) is a projective $\mathbb {Q}$-factorial dlt pair of dimension $\le 3$ such that $K_X+B\sim _\mathbb {R}0$ and $\kappa (X,B-B_\Phi )=0$. Then, $n(K_X+B)\sim 0.$

#### Proof

By Lemma 2.15, we may run an MMP on $-(K_X+B_\Phi ) \sim _\mathbb {R}B - B_\Phi$ which terminates with a good minimal model $X'$. Let $B'$ be the strict transform of *B* on $X'$. Since $\kappa (X,B-B_\Phi )=0$ and $B'-B'_\Phi$ is semi-ample, we see that $B'=B_{\Phi }'$ and, therefore, $K_{X'}+B_{\Phi }'\sim _\mathbb {R}0$. By [11, Proposition 6.4, Theorem 1.1] and [12, Theorem 2.14], there is a positive integer *n* depending only on $\Phi$ such that

$$\begin{aligned} n(K_{X'}+B')=n(K_{X'}+B_{\Phi }')\sim 0. \end{aligned}$$

It follows that $n(K_X+B)\sim 0$ as (*X*, *B*) and $(X',B')$ are crepant. $\square$

We will use the following results on the boundedness of complements.

#### Theorem 2.19

(cf. [47, Theorem 16]) Let *d*, *l* be two positive integers and $\Phi \subseteq [0,1]\cap \mathbb {Q}$ a hyperstandard set. Then, there exists a finite set of positive integers $\mathcal {N}$ divisible by *l* depending only on *d*, *l* and $\Phi$ satisfying the following.

Assume that $(X/Z\ni z,B)$ is a pair of dimension *d* such that *X* is of Fano type over *Z* and $(X/Z\ni z,B_{\mathcal {N}\_\Phi })$ has a klt $\mathbb {R}$-complement. Then, $(X/Z\ni z,B)$ is $\mathcal {N}$-complementary.

#### Theorem 2.20

([11, Theorem 1.3]) Let *l* be a positive integer. Then, there exists a finite set of positive integers $\mathcal {N}$ divisible by *l* depending on *l* satisfying the following. If $(X/Z\ni z,B)$ is an $\mathbb {R}$-complementary curve pair, then $(X/Z\ni z,B)$ is $\mathcal {N}$-complementary.

## Canonical Bundle Formulas

### Canonical Bundle Formulas

For the definition and basic properties of the canonical bundle formula, we refer the reader to [4, 15, 27, 30]. Briefly speaking, suppose that (*X*/*Z*, *B*) is a sub-pair and $\phi :X\rightarrow T$ is a contraction over *Z*, such that (*X*, *B*) is lc over the generic point of *T* and $K_X+B\sim _{\mathbb {R},T}0$. Then, there exist a uniquely determined $\mathbb {R}$-divisor $B_T$ and a nef over *Z* b-$\mathbb {R}$-divisor $\textbf{M}_\phi$ which is determined only up to $\mathbb {R}$-linear equivalence, such that $(T/Z,B_T+\textbf{M}_\phi )$ is a g-sub-pair and

$$\begin{aligned} K_X+B\sim _\mathbb {R}\phi ^*(K_T+B_T+\textbf{M}_{\phi ,T}). \end{aligned}$$

Here, *B* (respectively, $\textbf{M}_\phi$) is called the *discriminant part* (respectively, a *moduli part*) of the canonical bundle formula for (*X*/*Z*, *B*) over *T*. Moreover, if (*X*/*Z*, *B*) is an lc (respectively, klt) pair, then $(T/Z,B_T+\textbf{M}_\phi )$ is a glc (respectively, gklt) g-pair.

It is worthwhile to point out that $\textbf{M}_\phi$ only depends on (*X*, *B*) over the generic point of *T* (cf. [4, 3.4 (2)]), and there are many choices of $\textbf{M}_\phi$, some of which could behave badly. But we can always choose one with the required properties, e.g., Propositions 3.3 and 3.4.

#### Lemma 3.1

Notation as above.

1. Assume that (*X*, *B*) is a klt pair. Then, there exists a crepant model $(\tilde{T},B_{\tilde{T}}+\textbf{M}_{\phi }) \rightarrow (T,B_T+\textbf{M}_\phi )$ such that for any prime divisor $P\subseteq {\text {Supp}}B$ which is vertical over *T*, the image of *P* on $\tilde{T}$ is a prime divisor.
2. Suppose that there is an $\mathbb {R}$-divisor $G_T$ on *T* such that $(T,B_T+G_T+\textbf{M}_\phi )$ is a sub-glc g-sub-pair. If we let $G:=\phi ^*G_T$, then $(X,B+G)$ is sub-lc.

#### Proof

(1) According to [29, Theorem B.6] (cf. [1, Theorem 0.3], [31, Theorem 2], and [22, Theorem 2.8]), there exist birational morphisms $X'\rightarrow X$ and $T'\rightarrow T$ such that $X'\rightarrow T'$ is an equidimensional contraction. In particular, for any prime divisor $P\subseteq {\text {Supp}}B$ which is vertical over *T*, the image *Q* of *P* on $T'$ is a prime divisor. Moreover, by the canonical bundle formula, $a(Q,T,B_T+\textbf{M}_\phi )<1$ as $a(P,X,B)<1$. Since (*X*, *B*) is a klt pair, $(T,B_T+\textbf{M}_\phi )$ is a gklt g-pair. Therefore, (1) holds by [7, Lemma 4.6].

(2) Suppose on the contrary that $(X,B+G)$ is not sub-lc. Let $P''$ be a non-sub-lc place of $(X,B+G)$, i.e., $a(P'',X,B+G)<0$. It is clear that ${\text {Center}}_X(P'')\subseteq {\text {Supp}}G$ which is vertical over *T*. We can find birational morphisms $f:X''\rightarrow X$ and $g:T''\rightarrow T$ such that $X''\rightarrow T''$ is a contraction, $P''$ is a prime divisor on $X''$, and the image $Q''$ of $P''$ on $T''$ is a prime divisor (cf. [34, VI, Theorem 1.3]). We may write $K_{X''}+\Delta '':=f^*(K_X+B+G)\text { and }K_{T''}+\Delta _{T''}+\textbf{M}_{\phi ,T''}:=g^*(K_T+B_T+G_T+\textbf{M}_{\phi ,T})$ for some $\mathbb {R}$-divisors $\Delta ''$ and $\Delta _{T''}$. Then, $\Delta _{T''}$ is the discriminant part of the canonical bundle formula for $(X''/Z,\Delta '')$ over $T''$; see [43, Lemma 7.4 (ii)]. Since $(T,B_T+G_T+\textbf{M}_\phi )$ is sub-glc, ${\text {mult}}_{Q''}\Delta _{T''}\le 1$. By the definition of the canonical bundle formula, $(X'',\Delta '')$ is sub-lc over the generic point of $Q''$. In particular, ${\text {mult}}_{P''}\Delta ''=1-a(P'',X,B+G)\le 1$, a contradiction. $\square$

#### Lemma 3.2

Let *p* be a positive integer, (*X*, *B*) and $$(X,B')$$ two lc pairs, and $$\phi :X\rightarrow T$$ a contraction, such that $$B'\ge B$$, $$K_X+B\sim _{\mathbb {R},T}0$$, and $$K_X+B'\sim _{\mathbb {R},T}0$$. Let $$B_T$$ (respectively, $$B_T')$$ and $$\textbf{M}_{\phi }$$ be the discriminant part and a moduli part of the canonical bundle formula for (*X*, *B*) (respectively, $$(X,B'))$$ over *T*. If $$p(K_X+B)\sim p\phi ^*(K_T+B_T+\textbf{M}_{\phi ,T})$$, then $$p(K_X+B')\sim p\phi ^*(K_T+B_T'+\textbf{M}_{\phi ,T})$$.

#### Proof

Since $B'-B\ge 0$ and $B'-B\sim _{\mathbb {R},T}0$, $B'-B=\phi ^*H_T$ for some $\mathbb {R}$-Cartier $\mathbb {R}$-divisor $H_T$ on *T* by [12, Lemma 2.5]. Then, $B'_T=B_T+H_T$ by [43, Lemma 7.4 (ii)]. Therefore,

$$\begin{aligned} p(K_X+B')&=p(K_X+B)+p(B'-B)\sim p\phi ^*(K_T+B_T+\textbf{M}_{\phi ,T})+p\phi ^*H_T\\ {}&=p\phi ^*(K_T+B_T+H_T+\textbf{M}_{\phi ,T})=p\phi ^*(K_T+B_T'+\textbf{M}_{\phi ,T}). \end{aligned}$$

$$\square $$

#### Proposition 3.3

Let $\mathfrak {R}\subseteq [0,1]\cap \mathbb {Q}$ be a finite set, and $\Phi :=\Phi (\mathfrak {R})$. Then, there exist a positive integer *p* and a hyperstandard set $\Phi '\subseteq [0,1]\cap \mathbb {Q}$ depending only on $\Phi$ satisfying the following.

Assume that (*X*/*Z*, *B*) is an lc pair of dimension $\le 3$ and $\phi :X\rightarrow T$ is a contraction over *Z* such that $\dim T>0$, $B\in \Phi$, and $K_X+B\sim _{\mathbb {Q},T}0.$ Then, we can choose a moduli part $\textbf{M}_\phi$ of the canonical bundle formula for (*X*, *B*) over *T*, such that $B_T\in \Phi '$, $p\textbf{M}_\phi$ is b-Cartier, and

$$\begin{aligned} p(K_X+B)\sim p\phi ^*(K_T+B_T+\textbf{M}_{\phi ,T}), \end{aligned}$$

where $B_T$ is the discriminant part of the canonical bundle formula for (*X*, *B*) over *T*. Moreover, if $\dim T=\dim X-1$, then $p\textbf{M}_\phi$ is base point free over *Z*.

#### Proof

The result follows from [12, Proposition 3.1] and [16, Theorem 5.5]. $\square$

#### Proposition 3.4

Let $\Phi \subseteq [0,1]\cap \mathbb {Q}$ be a hyperstandard set. Then, there exists a positive integer *p* depending only on $\Phi$ satisfying the following.

Assume that (*X*/*Z*, *B*) is a klt threefold pair and $\phi :X\rightarrow T$ is a contraction over *Z*, such that $\dim T=1$, $B\in \Phi$, and $K_X+B\sim _{\mathbb {Q},Z}0$. Then, we can choose a moduli part $\textbf{M}_\phi$ of the canonical bundle formula for (*X*, *B*) over *T*, such that

$$\begin{aligned} p(K_X+B)\sim p\phi ^*(K_T+B_T+\textbf{M}_{\phi ,T}), \end{aligned}$$

and $p\textbf{M}_{\phi }$ is base point free over *Z*, where $B_T$ is the discriminant part of the canonical bundle formula for (*X*, *B*) over *T*.

#### Proof

If $\dim Z=1$, then $T=Z$. It follows that $p_1\textbf{M}_{\phi ,T}$ is Cartier and thus $p_1\textbf{M}_{\phi ,T}\sim _{Z}0$, where $p_1$ is given by Proposition 3.3 depending only on $\Phi$, and $\textbf{M}_\phi$ is a moduli part chosen as in Proposition 3.3. Therefore, in what follows, we may assume that $\dim Z=0$, i.e., *Z* is a point.

If $K_T\not \equiv 0$, then $T=\mathbb {P}^1$. Let $\textbf{M}_\phi$ be a moduli part chosen as in Proposition 3.3. Then, $p_1\textbf{M}_{\phi ,T}$ is base point free.

Now, assume that $K_T\equiv 0$ and in particular, $B_T=0$. Let *F* be a general fiber of $X\rightarrow T$ and $K_F+B_F:=(K_X+B)|_F\sim _\mathbb {Q}0$. According to [11, Proposition 6.4 and Theorem 1.1], $r(K_F+B_F)\sim 0$ for some positive integer *r* depending only on $\Phi$. Then, there exist a rational function $\alpha \in K(X)$ and an $\mathbb {R}$-Cartier $\mathbb {R}$-divisor *L* on *T* such that $K_X + B + \frac{1}{r}(\alpha ) = \phi ^*L$. Let $\textbf{M}_{\phi ,T} = L-K_T-B_T$. Then, $r(K_X+B)\sim r\phi ^*(K_T+B_T+\textbf{M}_{\phi ,T})$. Let $b_F$ be the second Betti number of a smooth model of the index one cover of *F*. By Lemma 2.8, there exists a positive real number $\epsilon$ which only depends on $\Phi$ such that $(F,B_F)$ is $\epsilon$-lc. If $B_F\ne 0$, then *F* belongs to a bounded family by [2, Theorem 6.9], and hence $b_F$ has an upper bound. If $B_F=0$, then $K_F\sim _\mathbb {Q}0$, and hence $b_F \le 22$ by the classification of surfaces. Therefore, by [17, Theorem 1.2], there exists a positive integer $p_2$ depending only on $b_F$ such that $p_2\textbf{M}_{\phi ,T}\sim 0$.

We conclude that $p:=p_1p_2r$ has the required property. $\square$

### Lifting Complements

Now, we turn to the following technical statement on lifting complements via the canonical bundle formula.

#### Proposition 3.5

Let *p* and *n* be two positive integers such that $p\mid n$. Let (*X*/*Z*, *B*) be an lc pair and $\phi :X\rightarrow T$ a contraction over *Z* such that $\dim T>0$ and $K_X+B\sim _{\mathbb {R},T}0$. Let $B_T$ and $\textbf{M}_{\phi }$ be the discriminant part and a moduli part of the canonical bundle formula for (*X*, *B*) over *T*, such that $p(K_{X}+B)\sim p\phi ^*(K_{T}+B_{T}+\textbf{M}_{\phi ,T})$ and $p \textbf{M}_{\phi }$ is b-Cartier. Let $(T',B_{T'}+\textbf{M}_{\phi }) \rightarrow (T,B_T+\textbf{M}_{\phi })$ be a crepant model and $M_{T'}$ an effective $\mathbb {Q}$-divisor on $T'$, such that

1. for any prime divisor $P\subseteq {\text {Supp}}B$ which is vertical over *T*, the image of *P* on $T'$ is a prime divisor,
2. $$pM_{T'}\sim _Z p\textbf{M}_{\phi ,T'}$$ and $$M_{T'}\wedge B_{T'}=0$$, and
3. $(T'/Z\ni z,B_{T'}+M_{T'})$ is *n*-complementary for some $z\in Z$.

Then, $(X/Z\ni z,B)$ is also *n*-complementary.

#### Proof

Let $X'$ be the normalization of the main component of $X\times _{T}T'$. Denote by $f:X'\rightarrow X$ and $\phi ':X'\rightarrow T'$ the induced morphisms. We may write $K_{X'}+B'=f^*(K_X+B)$ for some $\mathbb {R}$-divisor $B'$. Note that by our assumption, we have

$$\begin{aligned} p(K_{X'}+B')\sim p\phi '^*(K_{T'}+B_{T'}+\textbf{M}_{\phi ,T'})\sim _Z p\phi '^*(K_{T'}+B_{T'}+M_{T'}). \end{aligned}$$

Let $(T'/Z\ni z,B_{T'}^++M_{T'})$ be an *n*-complement of $(T'/Z\ni z,B_{T'}+M_{T'})$. We remark that as $p\mid n$, $B^+_{T'}\ge 0$. Possibly shrinking *Z* near *z*, we may assume that

$$\begin{aligned} n(K_{T'}+B_{T'}^++M_{T'})\sim _Z0. \end{aligned}$$

Let $B'^+:=B'+\phi '^*(B_{T'}^+-B_{T'})$ and $B^+:=f_*B'^+$. We claim that $(X/Z\ni z,B^+)$ is an *n*-complement of $(X/Z\ni z,B)$. Indeed, we have

$$\begin{aligned} n(K_{X'}+B'^+)&=n(K_{X'}+B')+n(B'^+-B')\\ {}&\sim _Z n\phi '^*(K_{T'}+B_{T'}+M_{T'})+n\phi '^*(B_{T'}^+-B_{T'})\\ {}&= n\phi '^*(K_{T'}+B^+_{T'}+M_{T'})\sim _Z0. \end{aligned}$$

Hence, $n(K_X+B^+)\sim _Z0$. According to Lemma 3.1 (2), the sub-pair $(X'/Z\ni z,B'^{+})$ is sub-lc, and thus $(X/Z\ni z,B^{+})$ is also sub-lc. It suffices to prove that

$$\begin{aligned} nB^+\ge \lfloor (n+1)\{B\}\rfloor +n\lfloor B\rfloor . \end{aligned}$$

Let $P\subseteq {\text {Supp}}B^+$ be a prime divisor. If *P* is horizontal over *T*, then ${\text {mult}}_PB^+={\text {mult}}_PB$ and there is nothing to prove. Therefore, we may assume that *Q*, the image of *P* on $T'$, is a prime divisor. Let $b_P:={\text {mult}}_PB$, $b_P^+:={\text {mult}}_PB^+,b_Q:={\text {mult}}_QB_{T'}$, $b_Q^+:={\text {mult}}_QB_{T'}^+$, and $m_Q:={\text {mult}}_P\phi '^*Q$ over the generic point of *Q*. It is clear that $b_Q^+\ge 0$ as $B_{T'}^+\ge 0$. By construction,

$$\begin{aligned} b_P^+=b_P+(b_Q^+-b_Q)m_Q. \end{aligned}$$

Hence,

$$\begin{aligned} r_{PQ}:=b_P+(1-b_Q)m_Q=b_P^++(1-b_Q^+)m_Q\in \frac{1}{n}\mathbb {Z}_{\ge 0}. \end{aligned}$$

Moreover, as $1-b_Q$ is the lc threshold of $\phi '^*Q$ with respect to $(X',B')$ over the generic point of *Q*, we know $r_{PQ}\le 1$. If $b_Q=1$, then $b_Q^+=1$ and thus $b_P=b_P^+.$ If $b_P=1$, then $r_{PQ}=b_Q=1$ and thus $b_P^+=1$. Hence, we may assume that $b_Q<1$ and $b_P<1$. Since $b_P=b_P^+- (b_Q^+-b_Q)m_Q$ and $nb_Q^+\ge \lfloor (n+1)b_Q\rfloor ,$ we can see that

$$\begin{aligned} \lfloor (n+1)b_P\rfloor&=\lfloor (n+1)b_P^++(n+1)(b_Q-b_Q^+)m_Q\rfloor \\&=nb_P^++\lfloor b_P^++((n+1)b_Q-nb_Q^+)m_Q-b_Q^+m_Q\rfloor \\&\le nb_P^++\lfloor b_P^++\{(n+1)b_Q\}m_Q-b_Q^+m_Q\rfloor \\&=nb_P^+, \end{aligned}$$

where the last equality holds as

$$\begin{aligned} b_P^++\{(n+1)b_Q\}m_Q-b_Q^+m_Q<b_P^++m_Q-b_Q^+m_Q=r_{PQ}\le 1. \end{aligned}$$

We finish the proof. $\square$

## Boundedness of Complements for sdlt Curves

### Definition 4.1

We say *X* is a *semismooth curve* if *X* is a reduced scheme of dimension 1, every irreducible component of *X* is normal, and all of its singularities are simple normal crossing points.

Let *X* be a semismooth curve, and let $B\ge 0$ be an $\mathbb {R}$-divisor on *X*. We say (*X*, *B*) is *sdlt* if *B* is supported in the smooth locus of *X* and $\left\lfloor {B}\right\rfloor \le 1$.

### Definition 4.2

Let *X* be a semismooth curve, and $B\ge 0$ an $\mathbb {R}$-divisor on *X*, such that (*X*, *B*) is sdlt. We say that $(X,B^+)$ is an *n*-*semi-complement* of (*X*, *B*), if

1. $$(X,B^+)$$ is sdlt,
2. $$nB^+\ge n\lfloor B\rfloor +\lfloor (n+1)\{B\}\rfloor $$, and
3. $$n(K_X+B^+)\sim 0$$.

Moreover, we say $(X,B^+)$ is *monotonic* if we additionally have $B^{+}\ge B$.

The following theorem is a generalization of [45, 5.2.2] and [33, 19.4 Theorem] where the case $l=1$ is proved.

### Theorem 4.3

Let *l* be a positive integer. Then, there exists a finite set of positive integers $\mathcal {N}_\mathrm{{sdlt}}$ divisible by *l* depending only on *l* satisfying the following.

Assume that *X* is a semismooth curve, connected but not necessarily complete, and $B\ge 0$ is an $\mathbb {R}$-divisor on *X*, such that

1. (*X*, *B*) is sdlt,
2. *X* has at least one complete component,
3. each incomplete component of *X* does not meet any other incomplete component of *X*,
4. the union of the complete components of *X* is connected, and
5. $-(K_X+B)$ is nef on each complete component of *X*.

Then, there exists an *n*-semi-complement $(X,B^+)$ of (*X*, *B*) in a neighborhood of the union of the complete components of *X* for some $n\in \mathcal {N}_\mathrm{{sdlt}}$.

### Proof

Let $X_0$ be a complete component of *X*, and let $\{P_1,\ldots ,P_k\}:=X_0\cap {\text {Sing}}X$. Then, $\deg (K_X|_{X_0})=2g-2+k$, where *g* is the genus of $X_0$. Since $\deg (K_X|_{X_0})\le 0,$ there are four possibilities:

(i) $$g=1,k=0,\deg (K_X|_{X_0})=0$$,
(ii) $$g=0,k=2,\deg (K_X|_{X_0})=0$$,
(iii) $$g=0,k=1,\deg (K_X|_{X_0})=-1$$,
(iv) $$g=0,k=0,\deg (K_X|_{X_0})=-2$$.

We remark that *B* could not meet the components of type (i) or (ii) as $\deg (K_X|_{X_0})=0$.

If $X_0$ is of type (i), then $X=X_0$ and $B=0$. In this case, (*X*, *B*) is *l*-complementary.

If $X_0$ is of type (iv), then $X=X_0$ and $X\cong \mathbb {P}^1$. By Theorem 2.20, there exists a finite set of positive integers $\mathcal {N}'$ divisible by *l* depending only on *l*, such that (*X*, *B*) is $\mathcal {N}'$-complementary.

Now, suppose that any complete component of *X* is either of type (ii) or of type (iii). Note that each component of type (ii) (respectively, type (iii)) can only meet other components at two points (respectively, one point). By assumptions (3) and (4), the entire curve *X* must form a chain or a cycle. If *X* is a cycle, then $B=0$ and $K_X \sim 0$ by Lemma 4.4. Otherwise, by Lemma 4.5, it suffices to construct $B^+$ such that $(X, B^+)$ is an *n*-complement of (*X*, *B*) on each component of *X*. Note that possibly shrinking *X* near the union of the complete components, for any positive integer *n*, (*X*, *B*) is an *n*-complement of itself on each incomplete component and each complete component of type (ii). Since *X* has at most two complete components of type (iii), by Lemma 4.6 there exists a finite set of positive integers $\mathcal {N}''$ divisible by *l* depending only on *l*, such that (*X*, *B*) has an *n*-complement on each complete component of type (iii) for some $n\in \mathcal {N}''$. Therefore, by Lemma 4.5, (*X*, *B*) has an *n*-semi-complement for some $n\in \mathcal {N}''$.

Let $\mathcal {N}_\mathrm{{sdlt}}:=\mathcal {N}'\cup \mathcal {N}''$ and we are done. $\square$

### Lemma 4.4

Let $X = \bigcup _{i=1}^m X_i$ be a semismooth curve which is a cycle of irreducible curves $X_i$. Suppose that $X_i \cong \mathbb {P}^1$ for any $1 \le i \le m$. Then, $K_X \sim 0$.

### Proof

For each integer $m\ge j \ge 2$, we construct a semismooth curve $Y_j$ in a smooth projective surface $S_{j}$ such that $Y_j$ is a cycle of *j* complete rational curves and $K_{S_j}+Y_j \sim 0$, in particular, $K_{Y_j} = (K_{S_j} + Y_j)|_{Y_j} \sim 0$. Let $Y_2 \subseteq \mathbb {P}^2 =: S_2$ be the union of a line and a conic which is semismooth. Then, $K_{S_2} + Y_2 \sim 0$ and thus $K_{Y_2} = (K_{S_2} + Y_2)|_{Y_2} \sim 0$. Suppose that we have constructed a semismooth curve $Y_{j-1}$ contained in a smooth projective surface $S_{j-1}$, such that $Y_{j-1}$ is a cycle of $j-1$ complete rational curves and $K_{S_{j-1}} + Y_{j-1} \sim 0$. Let $\pi _j: S_j \rightarrow S_{j-1}$ be the blow-up of $S_{j-1}$ at one snc point of $Y_{j-1}$, and $E_j$ the exceptional divisor of $\pi _j$. Let $Y_j = (\pi _j)_*^{-1} Y_{j-1} \cup E_j$. Then, we get a semismooth curve $Y_j \subseteq S_j$, which is a cycle of *j* complete rational curves, such that $K_{S_j} + Y_j \sim 0$. Since *X* is analytically isomorphic to $Y_m$, by [28, Appendix B, Theorem 2.1], $K_{Y_m}\sim 0$ implies $K_X \sim 0$. $\square$

### Lemma 4.5

Let $X = \bigcup _{i=1}^m X_i$ be a semismooth curve which is a chain of irreducible curves $X_i$. Suppose that *D* is an $\mathbb {R}$-divisor on *X*, supported in the smooth locus of *X*, such that $D|_{X_i} \sim 0$ for any $1 \le i \le m$. Then, $D \sim 0$.

### Proof

Let $X^{(i)} := \bigcup _{j=1}^i X_j$ for $1 \le i \le m$, and $P_i := X_i \cap X_{i+1} = X^{(i)} \cap X_{i+1}$ for $1 \le i \le m-1$. We will prove by induction that $D|_{X^{(i)}} \sim 0$ for any $1\le i\le m$. Suppose that $D|_{X^{(i-1)}} \sim 0$ for some integer $i\ge 2$. Then, there exist a rational function $\alpha _{i-1}$ on $X^{(i-1)}$ and a rational function $\beta _i$ on $X_i$, such that $D|_{X^{(i-1)}} = (\alpha _{i-1})$ and $D|_{X_i} = (\beta _i)$. Since $P_{i-1}$ is not contained in the support of *D*, $\alpha _{i-1}$ and $\beta _i$ are non-zero regular functions near $P_{i-1}$. Replacing $\beta _i$ by $\frac{\alpha _{i-1}(P_{i-1})}{\beta _i(P_{i-1})} \beta _i$, we may assume that $\alpha _{i-1}(P_{i-1}) = \beta _i(P_{i-1})$. Then, there exists a rational function $\alpha _i$ on $X^{(i)}$ such that $\alpha _i|_{X^{(i-1)}} = \alpha _{i-1}$ and $\alpha _i|_{X_i} = \beta _i$. Hence, $D|_{X^{(i)}} = (\alpha _i)$, and thus $D|_{X^{(i)}} \sim 0$. Therefore, by induction we see that $D \sim 0$. $\square$

### Lemma 4.6

Let *l* be a positive integer. Then, there exists a finite set of positive integers $\mathcal {N}''$ divisible by *l* depending only on *l* satisfying the following.

Assume that $\{a_i\}_{i=1}^{k}$ and $\{b_i\}_{i=1}^{k}$ are two sequences of non-negative real numbers, such that $\sum _{i=1}^{k} a_i\le 1$ and $\sum _{i=1}^{k} b_i\le 1$. Then, there exist positive integers $n\in \mathcal {N}''$ and $k'\ge k$, and two sequences of non-negative real numbers $\{a_i^{+}\}_{i=1}^{k'}$ and $\{b_i^{+}\}_{i=1}^{k'}$, such that

1. $$\sum _{i=1}^{k'} a_i^{+}=\sum _{i=1}^{k'} b_i^{+}=1$$, and
2. $na_i^{+}\ge n\left\lfloor { a_i}\right\rfloor +\left\lfloor {(n+1)\{a_i\}}\right\rfloor$ and $nb_i^{+}\ge n\left\lfloor { b_i}\right\rfloor +\left\lfloor {(n+1)\{b_i\}}\right\rfloor$ for any $1\le i\le k$.

### Proof

Without loss of generality, we may assume that $a_i,b_i<1$ for any *i*. Then, it suffices to prove

4.1 $$\begin{aligned} n-\sum _{i=1}^k\left\lfloor {(n+1)a_i}\right\rfloor \ge 0 \quad \text {and} \quad n-\sum _{i=1}^k\left\lfloor {(n+1)b_i}\right\rfloor \ge 0. \end{aligned}$$

For any positive integer *n* and non-negative real numbers *c*, *d*, we have

$$\begin{aligned} \lfloor (n+1)(c+d)\rfloor \ge \lfloor (n+1)c\rfloor + \lfloor (n+1)d\rfloor . \end{aligned}$$

Thus, possibly replacing $(a_i,a_j)$ by $(a_i+a_j,0)$ (respectively, $(b_i,b_j)$ by $(b_i+b_j,0)$), we may assume that $a_i+a_j\ge 1$ (respectively, $b_i+b_j\ge 1$) for any $i\ne j$. In particular, we may assume that $k=2$ and $a_1+a_2=b_1+b_2=1$.

By Dirichlet prime number theorem, there exist three distinct prime numbers $q_j$ such that $l\mid q_j-1$ for any $j\in \{1,2,3\}$. Let $n_j:=q_j-1$, and $\mathcal {N}'':=\{n_1,n_2,n_3\}$. We claim that there exists $n\in \mathcal {N}''$ satisfying (4.1). It suffices to show that both $(n_j+1)a_1$ and $(n_j+1)b_1$ are not integers for some $j\in \{1,2,3\}$. Otherwise, by the pigeonhole principle, we may assume that $a_1\in \frac{1}{n_j+1}\mathbb {Z}\cap [0,1)=\frac{1}{q_j}\mathbb {Z}\cap [0,1)$ for two indices $j\in \{1,2,3\}$, which is absurd. $\square$

### Proposition 4.7

Let *l* be a positive integer. Then, there exists a finite set of positive integers $\mathcal {N}$ divisible by *l* depending only on *l* satisfying the following.

Assume that $(X/Z\ni z,B)$ is a surface pair such that *z* is a closed point, (*X*, *B*) is dlt, $S:=\left\lfloor {B}\right\rfloor \ne 0$, $B-S$ is big over *Z* and $K_X+B\sim _{\mathbb {R},Z}0$. Then, over a neighborhood of *z*, and $(S,B_S)$ has an *n*-semi-complement for some $n\in \mathcal {N}$, where $K_S+B_S:=(K_X+B)|_{S}$.

### Proof

Let $\mathcal {N}_\mathrm{{sdlt}}$ and $\mathcal {N}_1$ be finite sets of positive integers divisible by *l* given by Theorem 4.3 and Theorem 2.20, respectively, which only depend on *l*. We will show that $\mathcal {N}:=\mathcal {N}_\mathrm{{sdlt}}\cup \mathcal {N}_1$ has the required property.

It is clear that *S* is a semismooth curve, and $(S,B_S)$ is sdlt. We first show that *S* is connected over a neighborhood of *z*. Otherwise, there exists a contraction $\phi :X\rightarrow T$ to a curve *T* such that the general fiber *F* of $\phi$ is $\mathbb {P}^1$ and each connected component of *S* is horizontal over *T*; see Shokurov’s connectedness lemma [33, 17.4 Theorem] and [42, Propositions 3.3.1 and 3.3.2] (see also [45, 5.7 Connectedness lemma], [21, Corollary 1.3]). Note that if $\dim Z=1$, then we take $T=Z$. As $B-S$ is big over *Z*, $B-S$ is horizontal over *T* and $(B-S)|_F\ne \emptyset$. It follows that $(K_X+B)|_F=(K_X+S+B-S)|_F\not \sim _\mathbb {R}0$, a contradiction. Thus, *S* is connected over a neighborhood of *z*. Possibly shrinking *Z* near *z*, we may assume that $K_X+B\sim _\mathbb {R}0$ and thus $K_S+B_S$ is trivial on each complete component of *S*. If *S* has two irreducible incomplete components $S_1$ and $S_2$ that $S_1\cap S_2\ne \emptyset$ over any neighborhood of *z*, then by assumption, we have $B=S=S_1+S_2$ over a neighborhood of *z*. In this case, $B_S=0$ and $K_S\sim 0$ over a neighborhood of *z*. Now, we assume that each irreducible incomplete component of *S* does not meet any other irreducible incomplete component of *S*. By the classification of dlt surface pairs (cf. [36, Corollary 5.55]), over a neighborhood of *z*, either the support of $B_S$ lies in the union of the complete components of *S* or *S* is irreducible and its image on *Z* is also a curve. In the former case, $(S,B_S)$ has an *n*-semi-complement in a neighborhood of the union of complete component of *S* for some $n\in \mathcal {N}_\mathrm{{sdlt}}$ by Theorem 4.3. Therefore, over a neighborhood of *z*, $(S,B_S)$ has an *n*-semi-complement. In the latter case, the morphism from *S* to its image on *Z* is a contraction, then $(S,B_S)$ has an *n*-complement over a neighborhood of *z* for some $n\in \mathcal {N}_1$. This finishes the proof. $\square$

## Boundedness of Complements for Surfaces

### Conjecture 2.16 for Surfaces

In this subsection, we confirm Conjecture 2.16 for surfaces. For convenience, by (Theorem $*$)_d_ we mean Theorem $*$ in dimension *d*.

**Notation** $(\star )$. Let $\Phi _1\subseteq [0,1]\cap \mathbb {Q}$ be a hyperstandard set. Let $p=p(\Phi _1)$ be a positive integer given by (Proposition 3.3)_2_ which only depends on $\Phi _1$. Let $\mathcal {N}_2=\mathcal {N}_2(p)$ be a finite set of positive integers divisible by *p* given by Theorem 2.20 which only depends on *p*, and let $\Phi _2:=\Gamma (\mathcal {N}_2,\Phi _1)$.

#### Proposition 5.1

Under Notation $(\star )$, assume that (*X*/*Z*, *B*) is a $\mathbb {Q}$-factorial lc surface pair such that $K_X+B\sim _{\mathbb {R},Z}0$ and $\kappa (X/Z,B-B_{\Phi _2})+\dim Z=\kappa (X/Z,B-B_{\Phi _1})+\dim Z=1$. Then, $(X/Z\ni z,B)$ is $\mathcal {N}_2$-complementary for any closed point $z\in Z$.

#### Proof

By Lemma 2.15 we can run an MMP on $-(K_X+B_{\Phi _2})\sim _{\mathbb {R},Z}B-B_{\Phi _2}$ over *Z* and reach a good minimal model $\psi :X\rightarrow X'$ over *Z*, such that $-(K_{X'}+B_{\Phi _2}')$ is semi-ample over *Z*, where $D'$ denotes the strict transform of *D* on $X'$ for any $\mathbb {R}$-divisor *D* on *X*. Let $\pi ':X'\rightarrow Z'$ be the contraction defined by $-(K_{X'}+B_{\Phi _2}')$ over *Z*. By assumption, $\dim Z'=1$. Let $B_{Z'}^{(2)}$ and $\textbf{M}_{\pi '}$ be the discriminant and moduli parts of the canonical bundle formula for $(X',B_{\Phi _{2}}')$ over $Z'$ in Proposition 3.3.

#### Claim 5.2

$p\textbf{M}_{\pi '}$ is base point free over *Z* and

$$\begin{aligned} p(K_{X'}+B_{\Phi _{2}}')\sim p(\pi ')^*\big (K_{Z'}+B_{Z'}^{(2)}+\textbf{M}_{\pi ',Z'}\big ). \end{aligned}$$

Assume Claim 5.2. Then,

$$\begin{aligned} p\big (K_{X}+B^{(2)}\big ):=p\psi ^*\big (K_{X'}+B'_{\Phi _2}\big )\sim p (\pi '\circ \psi )^{*}\big (K_{Z'}+B_{Z'}^{(2)}+\textbf{M}_{\pi ',Z'}\big ). \end{aligned}$$

Note that since $\psi$ is $-(K_X+B_{\Phi _2})$-negative, $B^{(2)}\ge B_{\Phi _2}$. Since $K_{X'}+B'\sim _{\mathbb {R},Z}0$ and $B\ge B_{\Phi _2}$, there exists a boundary $B_{Z'}$ on $Z'$ such that the g-pair $(Z',B_{Z'}+\textbf{M}_{\pi '})$ is glc, $B_{Z'}\ge B_{Z'}^{(2)}$, and $K_{Z'}+B_{Z'}+\textbf{M}_{\pi ',Z'}\sim _{\mathbb {R},Z}0$. As $p\textbf{M}_{\pi '}$ is base point free over *Z*, we can pick an effective $\mathbb {Q}$-divisor $M_{Z'}$ on $Z'$ such that

$$\begin{aligned} pM_{Z'}\sim _Z p\textbf{M}_{\pi ',Z'},\ M_{Z'}\wedge B_{Z'}=0,\ \text {and }(Z',B_{Z'}+M_{Z'})\text { is lc}. \end{aligned}$$

In particular, $(Z'/Z\ni z,B_{Z'}+M_{Z'})$ is an $\mathbb {R}$-complement of $(Z'/Z\ni z,B_{Z'}^{(2)}+M_{Z'})$ for any $z\in Z$. Now, by our choice of $\mathcal {N}_2$, $(Z'/Z\ni z,B_{Z'}^{(2)}+M_{Z'})$ is $\mathcal {N}_2$-complementary. According to Proposition 3.5, $(X/Z\ni z,B^{(2)})$ is $\mathcal {N}_2$-complementary, and hence $(X/Z\ni z,B_{\Phi _2})$ is also $\mathcal {N}_2$-complementary as $B^{(2)}\ge B_{\Phi _2}$. Thus, $(X/Z\ni z,B)$ is $\mathcal {N}_2$-complementary by Lemma 2.14. Therefore, it suffices to prove Claim 5.2.

#### Proof of Claim 5.2

According to Lemma 2.15 again, we may run an MMP on $-(K_{X'}+B'_{\Phi _1})\sim _{\mathbb {R},Z'}B'_{\Phi _{2}}-B'_{\Phi _{1}}$ over $Z'$ and reach a good minimal model $X'\rightarrow X''$ over $Z'$, such that $B''_{\Phi _{2}}-B''_{\Phi _{1}}$ is semi-ample over $Z'$, where $D''$ denotes the strict transform of $D'$ on $X''$ for any $\mathbb {R}$-divisor $D'$ on $X'$. One can pick a positive real number $\epsilon$, such that $g:X\rightarrow X''$ is also an MMP on $B-B_{\Phi _{2}}+\epsilon (B_{\Phi _{2}}-B_{\Phi _{1}})$ over *Z*. Furthermore, we may assume that $B''-B_{\Phi _{2}}''+\epsilon (B_{\Phi _{2}}''-B_{\Phi _{1}}'')$ is semi-ample over *Z* by Lemma 2.6. 
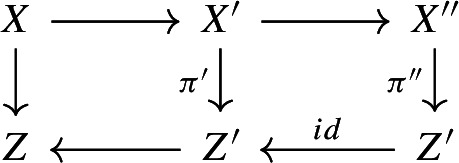
 By assumption,

$$\begin{aligned} \kappa (X/Z,B-B_{\Phi _{2}})=\kappa (X/Z,B-B_{\Phi _{2}}+\epsilon (B_{\Phi _{2}}-B_{\Phi _{1}})), \end{aligned}$$

and $B''-B''_{\Phi _{2}}+\epsilon (B''_{\Phi _{2}}-B''_{\Phi _{1}})\sim _{\mathbb {R},Z'}\epsilon (B''_{\Phi _{2}}-B^{''}_{\Phi _{1}})$. Hence, the natural morphism $\pi '':X''\rightarrow Z'$ is the contraction defined by $B''_{\Phi _{2}}-B^{''}_{\Phi _{1}}$ over $Z'$. In particular, we have

$$\begin{aligned} K_{X''}+B''_{\Phi _{1}}\sim _{\mathbb {R},Z'}0\quad \text {and}\quad K_{X''}+B''_{\Phi _{2}}\sim _{\mathbb {R},Z'}0. \end{aligned}$$

By Lemma 3.2 and Proposition 3.3, we see that $p\textbf{M}_{\pi '}$ is base point free, and

$$\begin{aligned} p(K_{X''}+B_{\Phi _2}'')\sim p(\pi '')^{*}\big (K_{Z'}+B_{Z'}^{(2)}+\textbf{M}_{\pi ',Z'}\big ). \end{aligned}$$

Since $X' \rightarrow X''$ is $(K_{X'}+B_{\Phi _2}')$-trivial, $(X',B_{\Phi _2}')$ and $(X'',B_{\Phi _2}'')$ are crepant. Therefore,

$$\begin{aligned} p(K_{X'}+B_{\Phi _2}')\sim p(\pi ')^{*}\big (K_{Z'}+B_{Z'}^{(2)}+\textbf{M}_{\pi ',Z'}\big ). \end{aligned}$$

We complete the proof. $\square$

#### Theorem 5.3

Let *l* be a positive integer and $\Phi \subseteq [0,1]\cap \mathbb {Q}$ a hyperstandard set. Then, there exists a finite set of positive integers $\mathcal {N}$ divisible by *l* depending only on *l* and $\Phi$ satisfying the following.

Assume that $(X/Z\ni z,B)$ is a surface pair such that $(X/Z\ni z,B_{\mathcal {N}\_\Phi })$ has a klt $\mathbb {R}$-complement. Then, $(X/Z\ni z,B)$ is $\mathcal {N}$-complementary.

#### Proof

Let $\mathcal {N}_1=\mathcal {N}_1(l,\Phi )$ be a finite set of positive integers divisible by *l* given by (Theorem 2.19)_2_ which only depends on *l* and $\Phi$, and let $\Phi _1:={\Gamma }(\mathcal {N}_1,\Phi )$. Let $p=p(l,\Phi _1)$ be a positive integer divisible by *l* given by (Proposition 3.3)_2_ which only depends on *l* and $\Phi _1$. Let $\mathcal {N}_2=\mathcal {N}_2(p)$ be a finite set of positive integers divisible by *p* given by Theorem 2.20 which only depends on *p*, and let $\Phi _2:=\Gamma (\mathcal {N}_1\cup \mathcal {N}_2,\Phi )$. Let $n_{CY}=n_{CY}(l, \Phi _2)$ be a positive integer divisible by *l* given by Lemma 2.18 which only depends on *l* and $\Phi _2$. We will show that the finite set $\mathcal {N}:=\mathcal {N}_1\cup \mathcal {N}_2\cup \{n_{CY}\}$ has the required property.

Possibly replacing *z* by a closed point of $\bar{z}$, we may assume that *z* is a closed point. Suppose that $(X/Z\ni z,B^+)$ is a klt $\mathbb {R}$-complement of $(X/Z\ni z,B_{\mathcal {N}\_\Phi })$. Possibly replacing (*X*, *B*) by a small $\mathbb {Q}$-factorialization of $(X,B^+)$ and shrinking *Z* near *z*, we may assume that (*X*, *B*) is $\mathbb {Q}$-factorial klt and $K_X+B\sim _{\mathbb {R},Z}0$. Since $B\ge B_{\Phi _2}\ge B_{\Phi _1}$,

$$\begin{aligned} 0\le \kappa (X/Z,B-B_{\Phi _2})+\dim Z\le \kappa (X/Z,B-B_{\Phi _1})+\dim Z\le 2. \end{aligned}$$

Therefore, we only need to consider the following three cases:

1. $$\kappa (X/Z,B-B_{\Phi _1})+\dim Z=2$$,
2. $$\kappa (X/Z,B-B_{\Phi _2})+\dim Z=\kappa (X/Z,B-B_{\Phi _1})+\dim Z=1$$, and
3. $$\kappa (X/Z,B-B_{\Phi _2})+\dim Z=0$$.

If $\kappa (X/Z,B-B_{\Phi _1})+\dim Z=2$, then *X* is of Fano type over *Z*. In this case $(X/Z\ni z,B)$ is $\mathcal {N}_1$-complementary by the choice of $\mathcal {N}_1$ (see Theorem 2.19). If $\kappa (X/Z,B-B_{\Phi _2})+\dim Z=\kappa (X/Z,B-B_{\Phi _1})+\dim Z=1$, then $(X/Z\ni z,B)$ is $\mathcal {N}_2$-complementary by Proposition 5.1. If $\kappa (X/Z,B-B_{\Phi _2})+\dim Z=0$, that is, $\dim Z=0$ and $\kappa (X,B-B_{\Phi _2})=0$, then one has

$$\begin{aligned} n_{CY}(K_X+B)\sim 0 \end{aligned}$$

by the choice of $n_{CY}$ (see Lemma 2.18). We finish the proof. $\square$

### Proof of Theorem 1.3

#### Proposition 5.4

(cf. [33, 16.7 Corollary]) Let $\mathfrak {R}\subseteq [0,1]\cap \mathbb {Q}$ be a finite set and $\Phi :=\Phi (\mathfrak {R})$. Then, there exists a hyperstandard set $\tilde{\Phi }$ depending only on $\Phi$ satisfying the following.

Assume that (*X*, *B*) is a dlt pair and $S:=\lfloor B\rfloor$. Let $K_S+B_S:=(K_X+B)|_S$. If $B\in \Phi$, then $B_S\in \tilde{\Phi }$, and if $B\in \Gamma (\{n\},\Phi )$ for some positive integer *n*, then $B_S\in {\Gamma }(\{n\},\tilde{\Phi })$.

#### Proof

Let

$$\begin{aligned} \tilde{\mathfrak {R}}:=\Big \{1-\sum (1-r_i)\ge 0\,\Big |\, r_i\in \mathfrak {R}\Big \},\quad \mathfrak {R}_1:= \bigg \{r-\frac{m}{n+1}\ge 0\,\bigg | \, r\in \mathfrak {R},m\in \mathbb {Z}_{\ge 0}\bigg \}, \end{aligned}$$

and

$$\begin{aligned} \tilde{\mathfrak {R}}_1&:=\Big \{1-\sum (1-r_i')\ge 0 \Big | \,r_i'\in \mathfrak {R}_1\Big \}\\&=\bigg \{1-\sum (1-r_i)-\frac{m}{n+1}\ge 0\,\bigg | \,r_i\in \mathfrak {R},m\in \mathbb {Z}_{\ge 0}\bigg \}. \end{aligned}$$

Let $\tilde{\Phi }:=\Phi (\tilde{\mathfrak {R}})$ and $\tilde{\Phi }_{1}:=\Phi (\tilde{\mathfrak {R}}_{1})$. It is clear that $\Phi (\mathfrak {R}_{1})={\Gamma }(\{n\},\Phi )$ and $\tilde{\Phi }_{1}={\Gamma }(\{n\},\tilde{\Phi })$. By [33, 16.7 Corollary], if $B\in \Phi$, then $B_{S}\in \tilde{\Phi }$, and if $B\in \Gamma (\{n\},\Phi )$, then $B_S\in {\Gamma }(\{n\},\tilde{\Phi })$. Therefore, $\tilde{\Phi }$ has the required property. $\square$

#### Proposition 5.5

Let $(X/Z\ni z, B)$ be a surface pair such that (*X*, *B*) is $\mathbb {Q}$-factorial dlt and $-(K_{X}+B)$ is nef and big over a neighborhood of *z*. Let $S:=\lfloor B\rfloor$ and $K_{S}+B_{S}:=(K_X+B)|_S$. Suppose that *S* intersects $X_z$, the fiber of $X\rightarrow Z$ over *z*, and $(S,B_{S})$ has a monotonic *n*-semi-complement $(S,B_{S}^{+})$ over a neighborhood of *z*. Then, $(X/Z\ni z, B)$ is *n*-complementary.

#### Proof

Possibly replacing *z* by a closed point of $\bar{z}$ and shrinking *Z* near *z*, we may assume that *z* is a closed point, (*X*, *B*) is $\mathbb {Q}$-factorial dlt, $-(K_{X}+B)$ is nef and big over *Z*, and $n(K_S+B_S^+)\sim 0$.

Let $g:W\rightarrow X$ be a log resolution of (*X*, *B*) such that *g* is an isomorphism over the snc locus of (*X*, *B*) (cf. [35, Theorem 10.45]), and let $S_W$ be the strict transform of *S* on *W*. Then, the induced morphism $g_{S_W}:=g|_{S_W}: S_W\rightarrow S$ is an isomorphism. We define

$$\begin{aligned} K_W+B_W:=g^{*}(K_X+B),\quad n\big (K_{S_W}+B_{S_W}^{+}\big ):=g_{S_W}^{*}\big (n(K_S+B_S^{+})\big )\sim 0, \end{aligned}$$

and

$$\begin{aligned} L_W:=\big \lceil -(n+1)(K_W+B_W)\big \rceil . \end{aligned}$$

Let $$\Delta _W:=B_W-S_W$$. Then,

$$\begin{aligned} K_{S_W}+B_{S_W}:=(K_W+B_W)|_{S_W}=K_{S_W}+\Delta _W|_{S_W}=g_{S_W}^{*}(K_S+B_S), \end{aligned}$$

and $$B_{S_W}<1$$ as $$\Delta _W<1$$.

Since $-(n+1)(K_W+B_W)$ is nef and big over *Z*, $R^1h_{*}(\mathcal {O}_{W}(K_W+L_W))=0$ by the relative Kawamata–Viehweg vanishing theorem for $\mathbb {R}$-divisors (cf. [19, Theorem 3.2.9]), where *h* is the induced morphism $W\rightarrow Z$. From the exact sequence,

$$\begin{aligned} 0\rightarrow \mathcal {O}_W(K_W+L_W)\rightarrow \mathcal {O}_W(K_W+S_W+L_W)\rightarrow \mathcal {O}_{S_W}(K_{S_W}+L_W|_{S_W})\rightarrow 0, \end{aligned}$$

we deduce that the induced map

$$\begin{aligned} H^0(W,K_W+S_W+L_W)\rightarrow H^0(S_W,K_{S_W}+L_W|_{S_W}) \end{aligned}$$

is surjective. Since $nB_{S_W}^{+}\in \mathbb {Z}$, $B_{S_W}<1$, and $B_{S_W}^{+}-B_{S_W}\ge 0$, we see that

$$\begin{aligned} G_{S_W}:=nB_{S_W}^{+}-\big \lfloor {(n+1)B_{S_W}}\big \rfloor \end{aligned}$$

is an effective integral divisor. We have

$$\begin{aligned} K_{S_W}+L_W|_{S_W}&=K_{S_W}+\big \lceil -(n+1)(K_{S_W}+B_{S_W})\big \rceil \\&=-nK_{S_W}-\big \lfloor {(n+1)B_{S_W}}\big \rfloor \sim G_{S_W}\ge 0. \end{aligned}$$

Thus, there exists $G_W\ge 0$ on *W* such that $G_W|_{S_W}=G_{S_W}$ and

$$\begin{aligned} G_W\sim K_W+S_W+L_W= -nK_W-nS_W-\lfloor (n+1)\Delta _W\rfloor . \end{aligned}$$

Let $$G:=g_{*}G_W$$, and

$$\begin{aligned} B^{+}:=S+\frac{1}{n}(\left\lfloor {(n+1)\{B\}}\right\rfloor +G). \end{aligned}$$

Then, we have

$$\begin{aligned} n(K_X+B^{+})= n(K_X+S)+\left\lfloor {(n+1)\{B\}}\right\rfloor +G\sim 0. \end{aligned}$$

It remains to show that $(X,B^{+})$ is lc over a neighborhood of *z*. Let *V* be the non-lc locus of $(X,B^{+})$. There exists a real number $a\in (0,1)$, such that the non-klt locus of $(X,aB^{+}+(1-a)B)$ is equal to $S\cup V$.

Since $g^{*}(K_X+B^+)|_{S_W}={g_{S_W}}^{*}(K_S+B_S^+)$, we have $(K_X+B^{+})|_S=K_{S}+B_{S}^{+}$ and $(K_X+aB^{+}+(1-a)B)|_{S}=K_S+aB_S^{+}+(1-a)B_S$. By inversion of adjunction (cf. [20, Theorem 1.4]), $(X,aB^{+}+(1-a)B)$ is lc near *S*. In particular, *S* is disjoint from *V*. Since

$$\begin{aligned} -(K_X+aB^{+}+(1-a)B)=-a(K_X+B^{+})-(1-a)(K_X+B) \end{aligned}$$

is nef and big over *Z*, by Shokurov–Kollár connectedness principle (cf. [33, 17.4 Theorem]), $(S\cup V)\cap X_z$ is connected. Recall that by assumption, $S\cap X_z\ne \emptyset$. Hence, $V\cap X_z=\emptyset$ and $(X,B^{+})$ is lc over a neighborhood of *z*. $\square$

#### Theorem 5.6

Let *l* be a positive integer and $\Phi \subseteq [0,1]\cap \mathbb {Q}$ a hyperstandard set. Then, there exists a finite set of positive integers $\mathcal {N}$ divisible by *l* depending only on *l* and $\Phi$ satisfying the following.

Assume that $(X/Z\ni z,B)$ is a surface pair such that $(X/Z\ni z,B_{\mathcal {N}\_\Phi })$ is $\mathbb {R}$-complementary. Then, $(X/Z\ni z,B)$ is $\mathcal {N}$-complementary.

#### Proof

Let $\tilde{\Phi }:=\Phi (\tilde{\mathfrak {R}})$ be the hyperstandard set associated to the finite set $\tilde{\mathfrak {R}}\subseteq [0,1]\cap \mathbb {Q}$ given by Proposition 5.4 which only depends on $\Phi$. Possibly replacing *l* by a multiple, we may assume that $l\tilde{\mathfrak {R}}\subseteq \mathbb {Z}_{\ge 0}$. Let $\mathcal {N}_0=\mathcal {N}_0(l, \Phi )$ be a finite set of positive integers divisible by *l* given by Theorem 5.3 which only depends on *l* and $\Phi$. Let $\mathcal {N}_1=\mathcal {N}_1(l)$ be a finite set of positive integers divisible by *l* given by Proposition 4.7 which only depends on *l*, and let $\Phi _1:=\Gamma (\mathcal {N}_1,\Phi )$. Let $p=p(l, \Phi _1)$ be a positive integer divisible by *l* given by (Proposition 3.3)_2_ which only depends on *l* and $\Phi _1$. Let $\mathcal {N}_2=\mathcal {N}_2(p)$ be a finite set of positive integers divisible by *p* given by Theorem 2.20 which only depends on *p*, and let $\Phi _2:=\Gamma (\mathcal {N}_1\cup \mathcal {N}_2,\Phi )$. Let $n_{CY} = n_{CY}(l, \Phi _2)$ be a positive integer divisible by *l* given by Lemma 2.18 which only depends on *l* and $\Phi _2$. We will show that the finite set $\mathcal {N}:=\mathcal {N}_0\cup \mathcal {N}_1\cup \mathcal {N}_2\cup \{n_{CY}\}$ has the required property.

Possibly replacing *z* by a closed point of $\bar{z}$, we may assume that *z* is a closed point. If $(X/Z\ni z,B_{\mathcal {N}\_\Phi })$ has a klt $\mathbb {R}$-complement, then so does $(X/Z\ni z,B_{{\mathcal {N}_0}\_\Phi })$, and hence $(X/Z\ni z,B)$ is $\mathcal {N}_0$-complementary by the choice of $\mathcal {N}_0$. Therefore, we may assume that $(X/Z\ni z,B_{\mathcal {N}\_\Phi })$ has an $\mathbb {R}$-complement $(X/Z\ni z,B_{\mathcal {N}\_\Phi }+G)$ which is not klt. Possibly replacing $(X/Z\ni z,B)$ by a $\mathbb {Q}$-factorial dlt modification of $(X/Z\ni z,B_{\mathcal {N}\_\Phi }+G)$ and shrinking *Z* near *z*, we may assume that (*X*, *B*) is $\mathbb {Q}$-factorial dlt, $K_X+B\sim _{\mathbb {R},Z}0$, and $S\cap X_z\ne \emptyset$, where $S:=\left\lfloor {B}\right\rfloor \ne 0$ and $X_z$ is the fiber of $X\rightarrow Z$ over *z*. Since $B\ge B_{\Phi _2}\ge B_{\Phi _1}\ge B_{\Phi }$, we have

$$\begin{aligned} 0&\le \kappa (X/Z,B-B_{\Phi _2})+\dim Z\le \kappa (X/Z,B-B_{\Phi _1})+\dim Z\\&\le \kappa (X/Z,B-B_{\Phi })+\dim Z\le 2. \end{aligned}$$

Therefore, we only need to consider the following three possibilities:

1. $$\kappa (X/Z,B-B_{\Phi _2})+\dim Z=0$$,
2. $$\kappa (X/Z,B-B_{\Phi _2})+\dim Z=\kappa (X/Z,B-B_{\Phi _1})+\dim Z=1$$, and
3. $$\kappa (X/Z,B-B_{\Phi _1})+\dim Z=\kappa (X/Z,B-B_{\Phi })+\dim Z=2$$.

If $\kappa (X/Z,B-B_{\Phi _2})+\dim Z=0$, then $n_{CY}(K_X+B)\sim 0$ by our choice of $n_{CY}$. If $\kappa (X/Z,B-B_{\Phi _2})+\dim Z=\kappa (X/Z,B-B_{\Phi _1})+\dim Z=1$, then $(X/Z\ni z,B)$ is $\mathcal {N}_2$-complementary by Proposition 5.1. Hence, in what follows we assume that $\kappa (X/Z,B-B_{\Phi _1})+\dim Z=\kappa (X/Z,B-B_{\Phi })+\dim Z=2$. We will show that $(X/Z\ni z,B)$ is $\mathcal {N}_1$-complementary.

In this case, both $B-B_{\Phi }$ and $B-B_{\Phi _{1}}$ are big over *Z*. Let $K_S+B_S:=(K_X+B)|_{S}\sim _{\mathbb {R},Z}0$. By Lemma 2.14 and the choice of $\mathcal {N}_1$, $(S,(B_S)_{n\_\tilde{\Phi }})$ has a monotonic *n*-semi-complement over a neighborhood of *z* for some $n\in \mathcal {N}_1$. Note that $B_{n\_\Phi }\in \Gamma (\{n\},\Phi )\subseteq \Gamma (\mathcal {N}_1,\Phi )$, $B_{n\_\Phi }\le B_{\Phi _{1}}$, and $B-B_{n\_\Phi }$ is big over *Z*. According to Lemma 2.15, we may run an MMP on $-(K_{X}+B_{n\_\Phi })\sim _{\mathbb {R},Z}B-B_{n\_\Phi }$ over *Z* and reach a minimal model $\psi :X\rightarrow X'$ over *Z*, such that $B'-B'_{n\_\Phi }$ is nef and big over *Z*, where $D'$ denotes the strict transform of *D* on $X'$ for any $\mathbb {R}$-divisor *D* on *X*. No component of *S* is contracted by $\psi$ and $\psi _S := \psi |_{S}:S\rightarrow S'$ is an isomorphism as $S\le B_{n\_\Phi }\le B$ and $\psi$ is $(K_X+B)$-trivial.

Since $-(K_X+B)-\psi ^{*}(-(K_{X'}+B'_{n\_\Phi }))$ is nef over $X'$, and $-B'+B'_{n\_\Phi }\le 0$, by the negativity lemma, $-(K_X+B)\le \psi ^{*}(-(K_{X'}+B'_{n\_\Phi }))$. Let

$$\begin{aligned} K_{S'}+B'_{n\_\Phi ,S'}:=(K_{X'}+B'_{n\_\Phi })|_{S'}. \end{aligned}$$

Note that $B'_{n\_\Phi ,S'}\in \Gamma (\{n\},\tilde{\Phi })$ by Proposition 5.4, and the support of $-(K_X+B)- \psi ^{*}(-(K_{X'}+B'_{n\_\Phi }))$ does not contain any component of *S*. Then,

$$\begin{aligned}&-(K_S+B_S)- \psi _{S}^{*}\big (-(K_{S'}+B'_{n\_\Phi ,S'})\big ) \\ {}&\quad =\big (-(K_X+B)- \psi ^{*}\big (-(K_{X'}+B'_{n\_\Phi })\big )\big )|_{S}\le 0. \end{aligned}$$

Let $B_S'$ be the strict transform of $B_S$ on $S'$. Since $(B_S')_{n\_\tilde{\Phi }},B'_{n\_\Phi ,S'}\in \Gamma (\{n\},\tilde{\Phi })$, and $B_S'\ge B'_{n\_\Phi ,S'}$, we deduce that $(B'_S)_{n\_\tilde{\Phi }}\ge B'_{n\_\Phi ,S'}$. Hence, $(S',B'_{n\_\Phi ,S'})$ has a monotonic *n*-semi-complement over a neighborhood of *z*. By Proposition 5.5 and Lemma 2.14, $(X'/Z\ni z,B'_{n\_\Phi })$ has a monotonic *n*-complement. Since $\psi$ is $-(K_X+B_{n\_\Phi })$-negative, $(X/Z\ni z,B_{n\_\Phi })$ has a monotonic *n*-complement $(X/Z\ni z,B^{+})$. By Lemma 2.14, $(X/Z\ni z,B^{+})$ is an *n*-complement of $(X/Z\ni z,B)$. $\square$

#### Proof of Theorem 1.3

The theorem follows by Theorem 5.6. $\square$

## Boundedness of Complements for Threefolds

We will prove the following theorem which is stronger than Theorem 1.1.

### Theorem 6.1

Let *l* be a positive integer and $\Phi \subseteq [0,1]\cap \mathbb {Q}$ a hyperstandard set. Then, there exists a finite set of positive integers $\mathcal {N}$ divisible by *l* depending only on *l* and $\Phi$ satisfying the following.

Assume that $(X/Z\ni z,B)$ is a threefold pair such that $(X/Z\ni z,B_{\mathcal {N}\_\Phi })$ has a klt $\mathbb {R}$-complement. Then, $(X/Z\ni z,B)$ is $\mathcal {N}$-complementary.

### Proof

Let $\mathcal {N}_1 = \mathcal {N}_1(l, \Phi )$ be a finite set of positive integers divisible by *l* given by (Theorem 2.19)_3_ which only depends on *l* and $\Phi$, and set $\Phi _1:={\Gamma }(\mathcal {N}_1,\Phi )$. Let $p_1 = p_1(l, \Phi _1)$ be a positive integer divisible by *l* given by (Proposition 3.3)_3_ and Proposition 3.4 which only depends on *l* and $\Phi _1$. Let $\mathcal {N}_2 = \mathcal {N}_2(p_1)$ be a finite set of positive integers divisible by $p_1$ given by Theorems 2.20 and 1.3 which only depends on $p_1$, and set $\Phi _2:=\Gamma (\mathcal {N}_1\cup \mathcal {N}_2,\Phi )$. Let $p_2 = p_2(p_1, \Phi _2)$ be a positive integer divisible by $p_1$ given by (Proposition 3.3)_3_ and Proposition 3.4 which only depends on $p_1$ and $\Phi _2$. Let $\mathcal {N}_3 = \mathcal {N}_3(p_2)$ be a finite set of positive integers divisible by $p_2$ given by Theorems 2.20 and 1.3 which only depends on $p_2$, and set $\Phi _3:=\Gamma (\mathcal {N}_1\cup \mathcal {N}_2\cup \mathcal {N}_3,\Phi )$. Let $n_{CY} = n_{CY}(l, \Phi _3)$ be a positive integer divisible by *l* given by Lemma 2.18 which only depends on *l* and $\Phi _3$. We will show that $\mathcal {N}:=\mathcal {N}_1\cup \mathcal {N}_2\cup \mathcal {N}_3\cup \{n_{CY}\}$ has the required property.

Replacing *z* by a closed point of $\bar{z}$, we may assume that *z* is a closed point. Possibly replacing $(X/Z\ni z,B)$ by a small $\mathbb {Q}$-factorialization of a klt $\mathbb {R}$-complement of $(X/Z\ni z,B_{\mathcal {N}\_\Phi })$ and shrinking *Z* near *z*, we may assume that (*X*, *B*) is $\mathbb {Q}$-factorial klt and $K_X+B\sim _{\mathbb {R},Z}0$.

If $\kappa (X/Z,B-B_{\Phi _1})+\dim Z=3$, then *X* is of Fano type over *Z*. In this case $(X/Z\ni z,B)$ is $\mathcal {N}_1$-complementary by the choice of $\mathcal {N}_1$ (see Theorem 2.19). If $\kappa (X/Z,B-B_{\Phi _3})+\dim Z=0$, then $n_{CY}(K_X+B)\sim 0$ by the choice of $n_{CY}$ (see Lemma 2.18). Therefore, in the following, we may assume that

$$\begin{aligned} 1&\le \kappa (X/Z,B-B_{\Phi _3})+\dim Z\le \kappa (X/Z,B-B_{\Phi _2})+\dim Z\\&\le \kappa (X/Z,B-B_{\Phi _1})+\dim Z\le 2. \end{aligned}$$

In particular, there exist integers $i,k\in \{1,2\}$ such that

$$\begin{aligned} \kappa (X/Z,B-B_{\Phi _i})+\dim Z=\kappa (X/Z,B-B_{\Phi _{i+1}})+\dim Z=k. \end{aligned}$$

We will show that $(X/Z\ni z,B_{\Phi _{i+1}})$ is $\mathcal {N}_{i+1}$-complementary and thus finish the proof by Lemma 2.14.

By Lemma 2.15, we can run an MMP on $-(K_X+B_{\Phi _{i+1}})\sim _{\mathbb {R},Z}B-B_{\Phi _{i+1}}$ over *Z* and reach a good minimal model $X\dashrightarrow X'$ over *Z*, such that $B'-B_{\Phi _{i+1}}'$ is semi-ample over *Z*, where $D'$ denotes the strict transform of *D* on $X'$ for any $\mathbb {R}$-divisor *D* on *X*. Let $\pi ':X'\rightarrow Z'$ be the contraction defined by $-(K_{X'}+B_{\Phi _{i+1}}')$ over *Z*. By assumption, $\dim Z'=k$. Let $B_{Z'}^{(i+1)}$ and $\textbf{M}_{\pi '}$ be the discriminant and moduli parts of the canonical bundle formula for $(X',B_{\Phi _{i+1}}')$ over $Z'$ in Proposition 3.3 (respectively, Proposition 3.4) if $k=2$ (respectively, $k=1$).

### Claim 6.2

$p_i\textbf{M}_{\pi '}$ is base point free over *Z*, and

$$\begin{aligned} p_{i}(K_{X'}+B_{\Phi _{i+1}}')\sim p_{i}(\pi ')^*\big (K_{Z'}+B_{Z'}^{(i+1)}+\textbf{M}_{\pi ',Z'}\big ). \end{aligned}$$

Assume Claim 6.2. As $X\dashrightarrow X'$ is an MMP on $-(K_X+B_{\Phi _{i+1}})$ over *Z*, for any prime divisor *P* on *X* which is exceptional over $X'$, we have

$$\begin{aligned} a(P,X',B_{\Phi _{i+1}}')<a(P,X,B_{\Phi _{i+1}})\le 1. \end{aligned}$$

Thus, we can find a crepant model $(\tilde{X},\tilde{B}^{(i+1)})$ of $(X',B_{\Phi _{i+1}}')$ such that $\tilde{X}$ and *X* are isomorphic in codimension one. 
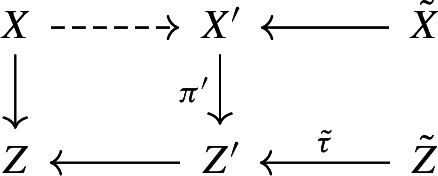
 It is clear that if $(\tilde{X}/Z\ni z,\tilde{B}^{(i+1)})$ is $\mathcal {N}_{i+1}$-complementary then so is $(X/Z\ni z,B_{\Phi _{i+1}})$. By Lemma 3.1, we may find a crepant model $(\tilde{Z},B^{(i+1)}_{\tilde{Z}}+\textbf{M}_{\pi '})\rightarrow (Z',B^{(i+1)}_{Z'}+\textbf{M}_{\pi '})$ such that for any prime divisor $P\subseteq {\text {Supp}}\tilde{B}^{(i+1)}$ which is vertical over $Z'$, the image of *P* on $\tilde{Z}$ is a prime divisor. As (*X*, *B*) is klt and $K_X+B\sim _{\mathbb {R},Z}0$, we may find a boundary $B_{\tilde{Z}}\ge B_{\tilde{Z}}^{(i+1)}$ on $\tilde{Z}$ such that $(\tilde{Z},B_{\tilde{Z}}+\textbf{M}_{\pi '})$ is gklt and $K_{\tilde{Z}}+B_{\tilde{Z}}+\textbf{M}_{\pi ',\tilde{Z}}\sim _{\mathbb {R},Z}0.$ Since $p_i\textbf{M}_{\pi '}$ is base point free over *Z*, we can pick an effective $\mathbb {Q}$-divisor $M_{\tilde{Z}}$ such that $p_iM_{\tilde{Z}}\sim _Z p_i\textbf{M}_{\pi ',\tilde{Z}}$, $M_{\tilde{Z}}\wedge B_{\tilde{Z}}^{(i+1)}=0$, and $(\tilde{Z},B_{\tilde{Z}}+M_{\tilde{Z}})$ is klt. By our choice of $\mathcal {N}_{i+1}$, $(\tilde{Z}/Z\ni z,B^{(i+1)}_{\tilde{Z}}+M_{\tilde{Z}})$ is *n*-complementary for some $n\in \mathcal {N}_{i+1}$. By Proposition 3.5, $(\tilde{X}/Z\ni z,\tilde{B}^{(i+1)})$ is also *n*-complementary. Therefore, it suffices to prove Claim 6.2.

### Proof of Claim 6.2

By Lemma 2.15, we may run an MMP on $-(K_{X'}+B_{\Phi _{i}}')\sim _{\mathbb {R},Z'}B'_{\Phi _{i+1}}-B'_{\Phi _{i}}$ over $Z'$ and reach a good minimal model $X'\dashrightarrow X''$ over $Z'$, such that $B''_{\Phi _{i+1}}-B''_{\Phi _{i}}$ is semi-ample over $Z'$, where $D''$ denotes the strict transform of $D'$ on $X''$ for any $\mathbb {R}$-divisor $D'$ on $X'$. Let $\pi '':X''\rightarrow Z''$ be the contraction defined by $B''_{\Phi _{i+1}}-B''_{\Phi _{i}}$ over $Z'$, and $\tau :Z''\rightarrow Z'$ the induced morphism. 
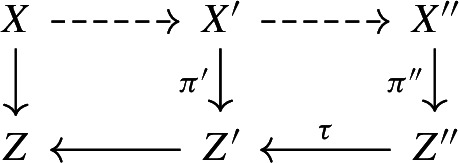
 We claim that $\tau$ is a birational morphism. In fact, one can pick a positive real number $\epsilon$, such that $B''-B_{\Phi _{i+1}}''+\epsilon (B_{\Phi _{i+1}}''-B_{\Phi _{i}}'')$ is semi-ample over *Z* (see Lemma 2.6) and that $X\dashrightarrow X''$ is also an MMP on $B-B_{\Phi _{i+1}}+\epsilon (B_{\Phi _{i+1}}-B_{\Phi _{i}})$ over *Z*. By assumption,

$$\begin{aligned} \kappa (X/Z,B-B_{\Phi _{i+1}})=\kappa \big (X/Z,B-B_{\Phi _{i+1}}+\epsilon (B_{\Phi _{i+1}}-B_{\Phi _{i}})\big ). \end{aligned}$$

Hence, we can see that $\tau :Z''\rightarrow Z'$ is birational.

By Lemma 3.2, Proposition 3.3 and the choice of $p_i$, there exists a gklt g-pair $(Z'',B_{Z''}^{(i+1)}+\textbf{M}_{\pi '})$ induced by the canonical bundle formula, such that $p_{i}\textbf{M}_{\pi '}$ is base point free over *Z* and

$$\begin{aligned} p_{i}(K_{X''}+B_{\Phi _{i+1}}'')\sim p_{i}(\pi '')^*\big (K_{Z''}+B_{Z''}^{(i+1)}+\textbf{M}_{\pi ',Z''}\big ). \end{aligned}$$

Moreover, it is clear that

$$\begin{aligned} K_{Z''}+B_{Z''}^{(i+1)}+\textbf{M}_{\pi ',Z''}=\tau ^*\big (K_{Z'}+B_{Z'}^{(i+1)}+\textbf{M}_{\pi ',Z'}\big ). \end{aligned}$$

Therefore,

$$\begin{aligned} p_{i}(K_{X'}+B_{\Phi _{i+1}}')\sim p_{i}(\pi ')^*\big (K_{Z'}+B_{Z'}^{(i+1)}+\textbf{M}_{\pi ',Z'}\big ) \end{aligned}$$

as $(X',B_{\Phi _{i+1}}')$ is crepant to $(X'',B_{\Phi _{i+1}}'')$. We finish the proof. $\square$

## Proof of Theorem 1.2

### Strictly Lc Calabi–Yau Pairs

#### Definition 7.1

We say that *X* is *of Calabi–Yau type* over *Z*, if $X\rightarrow Z$ is a contraction, and there is a boundary *C* such that (*X*, *C*) is klt and $K_X+C\sim _{\mathbb {R},Z}0$.

#### Lemma 7.2

Suppose that *X* is of Calabi–Yau type over *Z*. Assume that $(X,B^+)$ is lc, $K_X+B^+\sim _{\mathbb {R},Z}0$ for some boundary $B^+$, and $f: Y \rightarrow X$ is a projective birational morphism from a normal quasi-projective variety *Y*, such that $a(E_{i},X,B^+)<1$ for any prime exceptional divisor $E_{i}$ of *f*. Then, *Y* is of Calabi–Yau type over *Z*.

#### Proof

Since *X* is of Calabi–Yau type over *Z*, there exists a klt pair (*X*, *C*) such that $K_X+C\sim _{\mathbb {R},Z}0$. Let $D_{t}:=t B^{+}+(1-t)C$. Then, $(X,D_{t})$ is klt and $K_{X}+D_{t}\sim _{\mathbb {R},Z}0$ for any $t \in [0,1)$. We have

$$\begin{aligned} K_{Y}+B_{Y}^{+}+\sum _{i} (1-a_{i}^{+} ) E_{i}=f^{*} (K_{X}+B^{+} ) \end{aligned}$$

and

$$\begin{aligned} K_{Y}+C_{Y}+\sum _{i} (1-a_{i} ) E_{i}=f^{*} (K_{X}+C ), \end{aligned}$$

where $B_{Y}^{+}$ and $C_{Y}$ are the strict transforms of $B^{+}$ and *C* on *Y*, respectively, $a_{i}^{+}:=a (E_{i}, X, B^{+} )<1$, and $a_{i}:=a (E_{i}, X, C )$ for any *i*. Then,

$$\begin{aligned} \begin{aligned} K_{Y}+D_{t, Y}&:=f^{*} (K_{X}+D_{t} ) \\&= K_{Y}+t B_{Y}^{+}+(1-t) C_{Y}+\sum _{i} \big (t (1-a_{i}^{+} )+(1-t) (1-a_{i} ) \big ) E_{i} . \end{aligned} \end{aligned}$$

Pick $0<t_{0}<1$ such that $t_{0} (1-a_{i}^{+} )+ (1-t_{0} ) (1-a_{i} ) \ge 0$ for any *i*. Then, $(Y,D_{t_0,Y})$ is klt and $K_Y+D_{t_0,Y}\sim _{\mathbb {R},Z}0$. Therefore, *Y* is of Calabi–Yau type over *Z*. $\square$

#### Definition 7.3

(cf. [47, §11]) A pair $(X/Z\ni z,B)$ is called *strictly lc Calabi–Yau* if

1. $(X/Z\ni z,B)$ is an $\mathbb {R}$-complement of itself, and
2. for any $\mathbb {R}$-complement $(X/Z\ni z,B^+)$ of $(X/Z\ni z,B)$, $B^+=B$ over some neighborhood of *z*.

#### Remark 7.4

When $\dim Z=0$, (*X*, *B*) is strictly lc Calabi–Yau if and only if (1) holds.

#### Lemma 7.5

Assume that $(X/Z\ni z,B)$ is an $\mathbb {R}$-complement of itself. Then, $(X/Z\ni z,B)$ is strictly lc Calabi–Yau if and only if either $\dim z=\dim Z$ or $\bar{z}$ is the image of an lc center of (*X*, *B*) on *Z*.

#### Proof

First assume that $(X/Z\ni z,B)$ is strictly lc Calabi–Yau. Suppose that $\dim z<\dim Z$ and $\bar{z}$ is not the image of any lc center of (*X*, *B*). Possibly shrinking *Z* near *z*, we may find an ample divisor $H\ge 0$ such that $z\in {\text {Supp}}H$ and *H* does not contain the image of any lc center of (*X*, *B*). Pick a positive real number $\epsilon$, such that $(X/Z\ni z,B+\epsilon \pi ^*H)$ is lc and thus an $\mathbb {R}$-complement of $(X/Z\ni z,B)$. However, $B+\epsilon \pi ^*H\ne B$ over any neighborhood of *z*, a contradiction.

Now, we prove the converse direction. Assume that $(X/Z\ni z,B+G)$ is an $\mathbb {R}$-complement of $(X/Z\ni z,B)$ for some $G\ge 0$. Since $G\sim _\mathbb {R}0$ over some neighborhood of *z*, $G=\pi ^*L_Z$ for some $\mathbb {R}$-Cartier $\mathbb {R}$-divisor $L_Z$ on *Z* by [12, Lemma 2.5]. If $\dim z=\dim Z$, then $G=0$ over a neighborhood of *z*. If $\bar{z}$ is the image of some lc center of (*X*, *B*), then $z\notin {\text {Supp}}L_Z$ as $(X,B+G)$ is lc over a neighborhood of *z*. Therefore, in both cases, $(X/Z\ni z,B)$ is strictly lc Calabi–Yau. $\square$

#### Example 7.6

Let $\pi :X:=\mathbb {P}^1\times \mathbb {P}^1\rightarrow Z:=\mathbb {P}^1$, $z\in Z$ a closed point, and $L_1,L_2$ two sections. Then, over a neighborhood of *z*, we have $(X,L_1+L_2)$ is lc and $K_X+L_1+L_2\sim _{\mathbb {R},Z}0$. Since $K_X+L_1+L_2+\pi ^*z\sim _{\mathbb {R},Z}0$, $(X/Z\ni z,L_1+L_2)$ is not strictly lc Calabi–Yau.

#### Lemma 7.7

Suppose that $(X/Z\ni z,B)$ is strictly lc Calabi–Yau and $X\dashrightarrow X'$ is a birational contraction over *Z*. Let $B'$ be the strict transform of *B* on $X'$. Then, $(X'/Z\ni z,B')$ is strictly lc Calabi–Yau.

#### Proof

It follows from the definition of strictly lc Calabi–Yau and the fact that $(X,B)\dashrightarrow (X',B')$ is crepant over some neighborhood of *z*. $\square$

#### Proposition 7.8

Let $\Gamma \subseteq [0,1]\cap \mathbb {Q}$ be a DCC set. Then, there exists a positive integer *I* depending only on $\Gamma$ satisfying the following. If $(X/Z\ni z,B)$ is a strictly lc Calabi–Yau threefold pair such that $B\in \Gamma$, then $I(K_X+B)\sim 0$ over some neighborhood of *z*.

#### Proof

Possibly replacing $(X/Z\ni z,B)$ by a $\mathbb {Q}$-factorial dlt modification, we may assume that *X* is $\mathbb {Q}$-factorial. Since $(X/Z\ni z,B)$ is a strictly lc Calabi–Yau pair, by [26, Theorem 5.20], $B\in \Gamma '$ over a neighborhood of *z* for some finite subset $\Gamma '\subseteq \Gamma$ which only depends on $\Gamma$. According to [12, Theorem 2.14], we may find a positive integer *I* which only depends on $\Gamma '$ such that $(X/Z\ni z,B)$ has a monotonic *I*-complement $(X/Z\ni z,B+G)$ for some $G\ge 0$. By assumption, $G=0$ over some neighborhood of *z*. Thus,

$$\begin{aligned} I(K_X+B)=I(K_X+B+G)\sim 0 \end{aligned}$$

over some neighborhood of *z*. $\square$

### Proof of Theorem 1.2

We first show a special case of Theorem 1.2.

For convenience, we say a pair (*X*, *B*) is klt over a closed subset $Z_0\subseteq Z$, if $a(E,X,B)>0$ for any prime divisor *E* over *X* such that $\pi ({\text {Center}}_X(E))\subseteq Z_0$, where $\pi :X\rightarrow Z$ is a contraction. For two $\mathbb {R}$-divisors $D_1$ and $D_2$ on *X*, by $D_1\ge D_2$ (respectively, $D_1> D_2$) over $Z_0$, we mean that ${\text {mult}}_ED_1\ge {\text {mult}}_ED_2$ (respectively, ${\text {mult}}_ED_1>{\text {mult}}_ED_2$) for any prime divisor *E* on *X* with $\pi (E)\subseteq Z_0$. By $D_1\ge D_2$ over an open subset $U\subseteq Z$, we mean $D_1|_{\pi ^{-1}(U)}\ge D_2|_{\pi ^{-1}(U)}$.

#### Proposition 7.9

Let *I* be a positive integer. Assume that $\mathcal {N}$ is a finite set of positive integers divisible by *I* given by Theorem 1.1 which only depends on *I*.

Assume that $(X/Z\ni z,B)$ is an $\mathbb {R}$-complementary threefold pair such that *X* is of Calabi–Yau type over *Z*. Assume that there is a contraction $\pi ':X\rightarrow Z'$ over *Z*, and an open subset $U\subseteq Z'$, such that

1. $$IB\in \mathbb {Z}_{\ge 0}$$ over *U*,
2. (*X*, *B*) is klt over $Z'\setminus U$, and
3. $-(K_X+B)\sim _{\mathbb {R}}(\pi ')^*H'$ for some $\mathbb {R}$-divisor $H'$ which is ample over *Z*.

Then, $(X/Z\ni z,B)$ is $\mathcal {N}$-complementary.

#### Proof

Possibly shrinking *Z* near *z*, we may assume that (*X*, *B*) is lc. Set $N:=\max _{n\in \mathcal {N}}{n}$. We claim that there exists a boundary $B'$ on *X* such that

- $$(X,B')$$ is klt,
- $$K_X+B'\sim _{\mathbb {R},Z}0$$, and
- $B'\ge \frac{N}{N+1} B$ over *U* and $B'\ge B$ over $Z'\setminus U$.

Assume the claim holds. Then,

7.1 $$\begin{aligned} \lfloor (n+1)B'\rfloor \ge n\lfloor B\rfloor +\lfloor (n+1)\{B\}\rfloor \end{aligned}$$

for any $n\in \mathcal {N}$. By Theorem 1.1 and the construction of $\mathcal {N}$, $(X/Z\ni z,B')$ is *n*-complementary for some $n\in \mathcal {N}$. Thus, $(X/Z\ni z,B)$ is *n*-complementary by (7.1).

Therefore, it suffices to prove the claim. By assumption, we may find an effective $\mathbb {R}$-Cartier $\mathbb {R}$-divisor $H_1'\sim _{\mathbb {R},Z}H'$ such that $Z'\setminus U\subseteq {\text {Supp}}H_1'$ and $(X,B+H_1)$ is lc, where $H_1:=(\pi ')^*H_1'$. In particular, we have

$$\begin{aligned}{} & {} K_X+B+H_1\sim _{\mathbb {R},Z}0,\\ {}{} & {} B+H_1>B \quad \text {over }Z'\setminus U. \end{aligned}$$

Since *X* is of Calabi–Yau type over *Z*, there exists a boundary *C* such that (*X*, *C*) is klt and $K_X+C\sim _{\mathbb {R},Z}0$. Let $\delta \in (0,1)$ be a positive real number such that

$$\begin{aligned}{} & {} B':=(1-\delta )(B+H_1)+\delta C\ge \frac{N}{N+1}B\quad \text{ over } U,\\ {}{} & {} (1-\delta )(B+H_1)\ge B\quad \text{ over } Z'\setminus U. \end{aligned}$$

It is clear that $(X,B')$ is klt and $K_X+B'\sim _{\mathbb {R},Z}0$. This completes the proof. $\square$

#### Proof of Theorem 1.2

Let $I=I(l, \Gamma \cap \mathbb {Q})$ be a positive integer divisible by *l* given by Proposition 7.8 which only depends on *l* and $\Gamma \cap \mathbb {Q}$, and let $\Phi :=\Phi (\frac{1}{I}\mathbb {Z}\cap [0,1])$. Let $\mathcal {N}=\mathcal {N}(I)$ be a finite set of positive integers divisible by *I* given by Theorem 1.1 which only depends on *I*. We will show that $\mathcal {N}$ has the required property.

Possibly shrinking *Z* near *z*, we may assume that (*X*, *B*) is lc. By Lemma 7.2, we can replace (*X*, *B*) by a dlt modification and thus assume that (*X*, *B*) is $\mathbb {Q}$-factorial dlt. Suppose that $(X/Z\ni z,B^+)$ is an $\mathbb {R}$-complement of $(X/Z\ni z,B)$. Possibly replacing *z* by a closed point of $\bar{z}$ and shrinking *Z* near *z*, we may assume that *z* is a closed point, $(X,B^+)$ is lc, and $K_X+B^+\sim _{\mathbb {R},Z}0$. Write

$$\begin{aligned} -(K_X+B_{\mathcal {N}\_\Phi })\sim _{\mathbb {R},Z} B^+-B_{\mathcal {N}\_\Phi }=F+M, \end{aligned}$$

where $F:=N_\sigma (B^+-B_{\mathcal {N}\_\Phi }/Z)\ge 0$ and $M:=B^+-B_{\mathcal {N}\_\Phi }-F\ge 0$ (cf. [41, III, §4], [39, §3]). Note that *F* is well defined as $B^+-B_{\mathcal {N}\_\Phi }\ge 0$. Since *X* is of Calabi–Yau type over *Z*, there exists a boundary *C* such that (*X*, *C*) is klt and $K_X+C\sim _{\mathbb {R},Z}0$. Choose a positive real number $\epsilon _0$ such that $(X,C+\epsilon _0 M)$ is klt. We may run an MMP on $K_X+C+\epsilon _0M$ over *Z* and reach a good minimal model $X'$, such that $K_{X'}+C'+\epsilon _0M'$ is semi-ample over *Z*, where $D'$ denotes the strict transform of *D* on $X'$ for any $\mathbb {R}$-divisor *D* on *X*. Since

$$\begin{aligned} K_X+C+\epsilon _0M\sim _{\mathbb {R},Z}\epsilon _0M\sim _{\mathbb {R},Z}-\epsilon _0(K_X+B_{\mathcal {N}\_\Phi }+F), \end{aligned}$$

*X* and $X'$ are isomorphic in codimension one by [23, Lemma 2.4]. We also see that $-(K_{X'}+B'_{\mathcal {N}\_\Phi }+F')$ is semi-ample over *Z* and thus induces a contraction $\pi ':X'\rightarrow Z'$ over *Z*. In particular, there is an effective $\mathbb {R}$-divisor $H'$ on $Z'$ which is ample over *Z* such that $-(K_{X'}+B'_{\mathcal {N}\_\Phi }+F')\sim _{\mathbb {R}}(\pi ')^*H'$. Note that $(X',B^{+\prime })$ is lc and thus $(X',B_{\mathcal {N}\_\Phi }'+F')$ is also lc.

Let $\eta '$ be the generic point of $Z'$, and

$$\begin{aligned} \mathcal {Z}_\mathrm{{scy}}:=\{\eta '\} \cup \big \{z'\in Z' \,\big | \, \big (X'/Z'\ni z',B'_{\mathcal {N}\_\Phi }+F'\big )\text { is strictly lc Calabi--Yau}\big \}. \end{aligned}$$

By Lemma 7.5 and [3, Theorem 1.1], $\mathcal {Z}_\mathrm{{scy}}$ is a non-empty finite set.

#### Claim 7.10

We have $I(K_{X'}+B'_{\mathcal {N}\_\Phi }+F')\sim 0$ over a neighborhood of $z'$ for any $z'\in \mathcal {Z}_\mathrm{{scy}}$.

Assume Claim 7.10. By [12, Lemma 2.6], Lemma 7.5 and [3, Theorem 1.1], there exists an open subset $U\subseteq Z'$ such that

$$\begin{aligned} I\big (K_{X'}+B'_{\mathcal {N}\_\Phi }+F'\big )\sim 0\text { over }\{U\} \quad \text {and}\quad \big (X',B'_{\mathcal {N}\_\Phi }+F'\big )\text { is klt over }Z'\setminus U. \end{aligned}$$

In particular, $I(B'_{\mathcal {N}\_\Phi }+F')\in \mathbb {Z}_{\ge 0}$ over *U*. Recall that $-(K_{X'}+B'_{\mathcal {N}\_\Phi }+F')\sim _{\mathbb {R}}(\pi ')^*H'$ where $H'$ is ample over *Z*. By Proposition 7.9, $(X'/Z\ni z,B'_{\mathcal {N}\_\Phi }+F')$ is $\mathcal {N}$-complementary, and thus $(X'/Z\ni z,B')$ is also $\mathcal {N}$-complementary by Lemma 2.14. Moreover, as *X* and $X'$ are isomorphic in codimension one, $(X/Z\ni z,B)$ is $\mathcal {N}$-complementary. $\square$

#### Proof of Claim 7.10

We may pick a positive real number $\epsilon$ such that $(X',C'+\epsilon F')$ is klt and that $X\dashrightarrow X'$ is a sequence of steps of the $-(K_X+B_{\mathcal {N}\_\Phi }+(1-\epsilon )F)$-MMP over *Z*. Since

$$\begin{aligned} -\left( K_{X'}+B'_{\mathcal {N}\_\Phi }+(1-\epsilon )F'\right)&\sim _{\mathbb {R},Z'}\epsilon F'\sim _{\mathbb {R},Z'}K_{X'}+C'+\epsilon F', \end{aligned}$$

one can run an MMP on $-(K_{X'}+B'_{\mathcal {N}\_\Phi }+(1-\epsilon )F')$ over $Z'$. This MMP terminates with a model $X''$ on which $-(K_{X''}+B''_{\mathcal {N}\_\Phi }+(1-\epsilon )F'')$ is semi-ample over $Z'$, where $D''$ denotes the strict transform of $D'$ on $X''$ for any $\mathbb {R}$-divisor $D'$ on $X'$. 
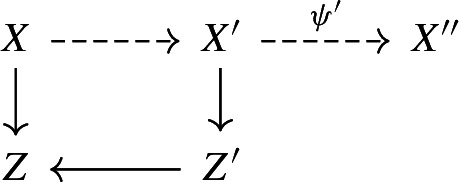
 For any positive real number $\epsilon '\ll \epsilon$, we infer that $\psi : X\dashrightarrow X''$ is also an MMP on $-(K_{X}+B_{\mathcal {N}\_\Phi }+(1-\epsilon ')F)$ over *Z*, and

$$\begin{aligned}&-\big (K_{X''}+B''_{\mathcal {N}\_\Phi }+(1-\epsilon ')F''\big )\\&\quad =-\bigg (1-\frac{\epsilon '}{\epsilon }\bigg )\big (K_{X''}+B''_{\mathcal {N}\_\Phi }+F''\big )- \frac{\epsilon '}{\epsilon }\big (K_{X''}+B''_{\mathcal {N}\_\Phi }+(1-\epsilon )F''\big ) \end{aligned}$$

is semi-ample over *Z* (see Lemma 2.6). In particular, $X''$ is a good minimal model of $-(K_{X}+B_{\mathcal {N}\_\Phi }+(1-\epsilon ')F)$ over *Z*. Since $N_\sigma (-(K_{X}+B_{\mathcal {N}\_\Phi }+(1-\epsilon ')F)/Z)=\epsilon ' F$ (cf. [41, III, 4.2 Lemma]), by [23, Lemma 2.4], *F* is contracted by $\psi$. Hence,

$$\begin{aligned} K_{X''}+B''_{\mathcal {N}\_\Phi }=\psi '_*\left( K_{X'}+B'_{\mathcal {N}\_\Phi }+F'\right) . \end{aligned}$$

If $\dim X'=\dim Z'$ and $z'=\eta '\in \mathcal {Z}_{\textrm{scy}}$, then $X'$ is smooth and $B^{+\prime }=0$ over a neighborhood of $\eta '$. Otherwise, by Lemma 7.7, we know that $({X''}/Z'\ni z',B''_{\mathcal {N}\_\Phi })$ is strictly lc Calabi–Yau for any $z'\in \mathcal {Z}_{\textrm{scy}}$, which implies that $B''_{\mathcal {N}\_\Phi }=B^{+\prime \prime }=B''\in \Gamma \cap \mathbb {Q}$ over a neighborhood of $z'$. We, therefore, see that $I(K_{X''}+B''_{\mathcal {N}\_\Phi })\sim 0$ over some neighborhood of $z'$ by our choice of *I*. Since $(X',B'_{\mathcal {N}\_\Phi }+F')$ is crepant to $(X'',B''_{\mathcal {N}\_\Phi })$, our claim holds. $\square$
